# Individualized dynamic prediction of survival with the presence of intermediate events

**DOI:** 10.1002/sim.8387

**Published:** 2019-10-30

**Authors:** Grigorios Papageorgiou, Mostafa M. Mokhles, Johanna J. M. Takkenberg, Dimitris Rizopoulos

**Affiliations:** ^1^ Department of Biostatistics Erasmus University Medical Centre Rotterdam The Netherlands; ^2^ Department of Cardiothoracic Surgery Erasmus University Medical Centre Rotterdam The Netherlands

**Keywords:** dynamic predictions, joint modeling, longitudinal data, survival data

## Abstract

Often, in follow‐up studies, patients experience intermediate events, such as reinterventions or adverse events, which directly affect the shapes of their longitudinal profiles. Our work is motivated by two studies in which such intermediate events have been recorded during follow‐up. In both studies, we are interested in the change of the longitudinal evolutions after the occurrence of the intermediate event and in utilizing this information to improve the accuracy of dynamic prediction of their risk. To achieve so, we propose a flexible joint modeling framework for longitudinal and time‐to‐event data, which includes features of the intermediate event as time‐varying covariates in both the longitudinal and survival submodels. We consider a set of joint models that postulate different effects of the intermediate event in the longitudinal profile and the risk of the clinical endpoint, with different formulations for the association structure while allowing its functional form to change after the occurrence of the intermediate event. Based on these models, we derive dynamic predictions of conditional survival probabilities which are adaptive to different scenarios with respect to the occurrence of the intermediate event. We evaluate the predictive accuracy of these predictions with a simulation study using the time‐dependent area under the receiver operating characteristic curve and the expected prediction error adjusted to our setting. The results suggest that accounting for the changes in the longitudinal profiles and the instantaneous risk for the clinical endpoint is important, and improves the accuracy of the dynamic predictions.

## 1 INTRODUCTION

Nowadays, there is great interest in the medical field for predictive tools that facilitate precision medicine. In the context of follow‐up studies, in which patients are monitored with several longitudinally measured parameters and biomarkers, physicians are interested in utilizing this information for predicting clinical endpoints. In this setting, joint models for longitudinal and survival outcomes provide a flexible framework to study the association between these outcomes and derive dynamic individualized predictions.^1^, ^2^, ^3^

The evaluation of the accuracy of these predictions obtained from joint models has gathered a lot of attention lately.^4^, ^5^, ^6^ An important observation that has been made is that the accuracy of the derived predictions is influenced by the appropriate modeling of the subject‐specific longitudinal profiles. In that regard, often, in follow‐up studies, intermediate events occur in some patients that directly affect the shapes of their longitudinal evolutions. These may include events that are either directly in the control of the investigators, such as additional reinterventions, or maybe not, such as adverse events that the patients may experience. While such intermediate events are common, very little work has been done in the direction of developing predictive tools that account for them and are adaptive to different scenarios with respect to their occurrence. To our knowledge, only Sène et al^7^ and Taylor et al^8^ investigated this topic in the context of prostate cancer recurrence and radiotherapy as an intermediate event. In their approach, however, they only considered the biomarker trajectories up to the occurrence of the intermediate event assuming extrapolation of the longitudinal profile thereafter. That is, changes in the shape of the longitudinal profile due to the occurrence of the intermediate event were not accounted for. Our goal is to show that utilizing the whole longitudinal trajectory, while capturing the changes to its shape due to the occurrence of intermediate events, can considerably improve the accuracy of such predictions.

**Figure 1:**
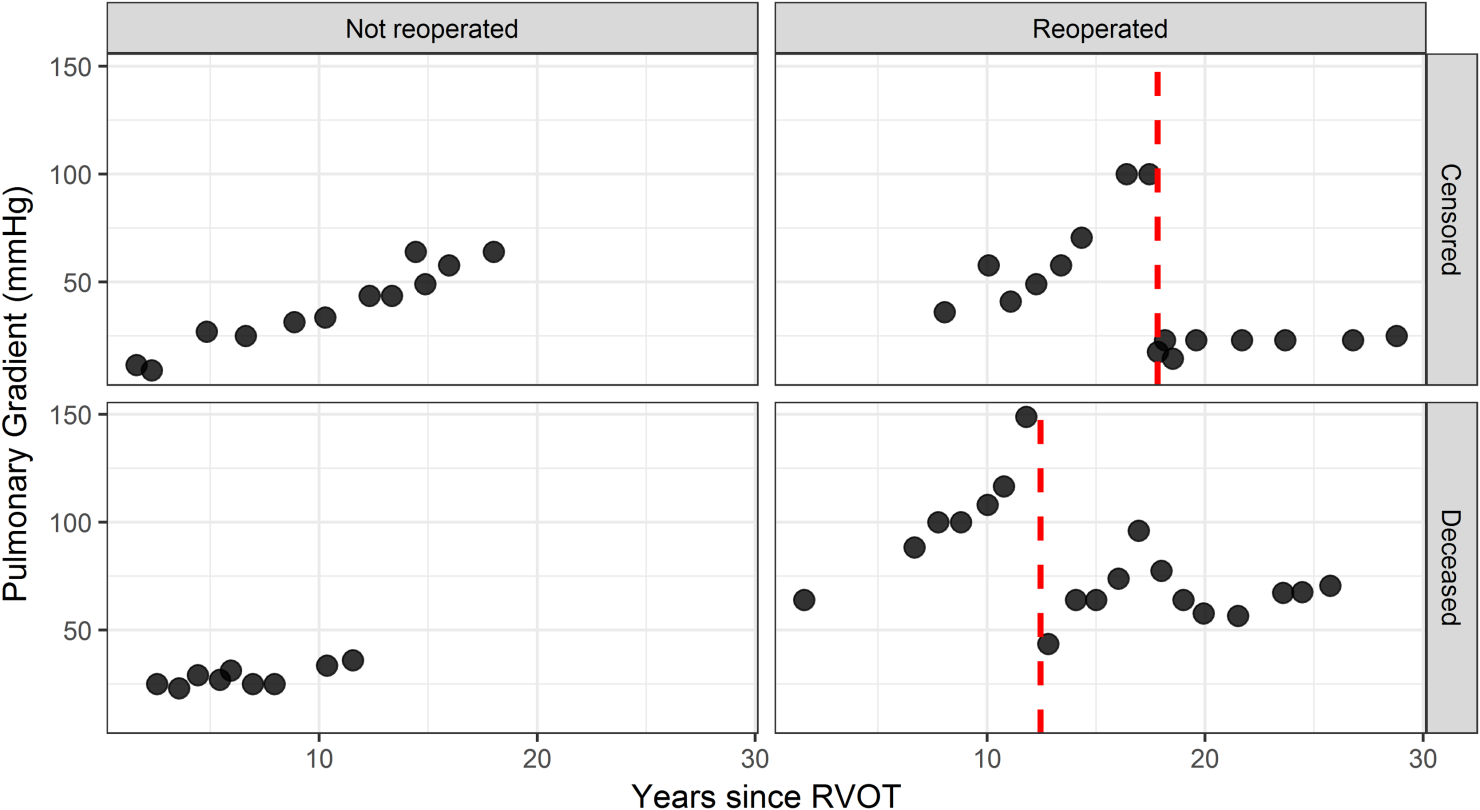
Pulmonary gradient profile of four randomly selected patients, one from each of the following categories: not reoperated and censored, not reoperated and deceased, reoperated and censored, reoperated and deceased. The vertical red dashed lines depict the time of reoperation. RVOT, right ventricular outflow tract [Colour figure can be viewed at http://wileyonlinelibrary.com]

**Figure 2:**
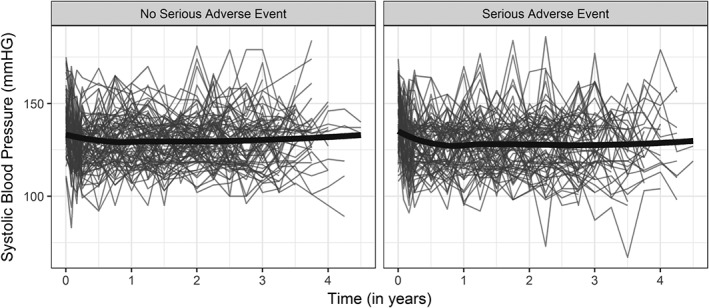
Individual evolutions and loess splines curves (solid thick lines) of systolic blood pressure for 80 randomly selected subjects who experienced a serious adverse event and for 80 randomly selected subjects who did not experience a serious adverse event

In our work, we are motivated by two studies in which such intermediate events have been recorded during follow‐up. The first study concerns 467 congenital heart‐diseased patients who underwent a right ventricular outflow tract reconstruction with a pulmonary valve and were followed‐up echocardiographically thereafter at the Department of Cardio‐Thoracic surgery of Erasmus University Medical Center. Death is considered as the study endpoint, while pulmonary gradient is the biomarker of interest, which is believed to be related to the risk of death. During follow‐up, 65(13.92%) were reoperated and received a pulmonary allograft. In Figure 1, the pulmonary gradient evolutions of four randomly selected patients, one from each combination between the reoperation and event status, are shown and it can be seen that when a patient is reoperated the pulmonary gradient drops. The interest in this study lies in the association between the pulmonary gradient and the risk of death, but the main focus is to study the impact of reoperation on the risk of death both directly and indirectly (ie, through its association with the pulmonary gradient) in order to develop predictive tools that can quantify the potential benefit of reoperation for future patients. The second study concerns 9361 subjects who participated in the SPRINT study.^9^ Subjects with increased cardiovascular risk, systolic blood pressure of 130mm Hg or higher but without diabetes, were randomized to intensive or standard treatment. The composite primary outcome was myocardial infarction, acute coronary syndromes, stroke, heart failure, or death from cardiovascular causes, while systolic blood pressure is the biomarker of interest which was repeatedly measured. During follow‐up, 3424(36.6%) experienced serious adverse events (SAEs). In Figure 2, the systolic blood pressure evolutions over time of the subjects who experienced SAEs and those who did not are depicted along with a loess curve for the average evolution over time for each group. The interest lies in assessing the impact of SAEs both in the systolic blood pressure evolution and the risk for the event of interest and exploiting it to derive individualized dynamic predictions for future patients with different scenarios with respect to the occurrence or not of SAEs. A more detailed description of the data sets is on the online supplementary material Sections S1.1 and S1.2.

In both studies, physicians are interested in obtaining predictions of the respective clinical endpoints. However, to provide predictions of adequate accuracy, it will be required to carefully model the subject‐specific longitudinal trajectories. Borrowing ideas from piecewise regression models, we achieve this by explicitly introducing the occurrence of these intermediate events as binary time‐varying covariates in the specification of both the longitudinal and survival submodels of the joint model. The regression coefficient associated with this covariate can then capture changes, due to the occurrence of the intermediate event, in both the biomarker trajectory and the hazard for the event of interest. Furthermore, we allow features of the biomarker trajectory, such as the rate of change to differ after the occurrence of the intermediate event. This allows us to estimate the impact of intermediate events as well as their specific features, which then can be utilized in deriving dynamic predictions for a future patient under different scenarios, eg, how the risk of a patient changes assuming different treatment strategies, such as no reintervention, reintervention now, or reintervention at a later time point.

The rest of this paper is structured as follows. Section 2 describes the formulation of the joint model in the presence of intermediate events. Section 3 presents the individualized dynamic predictions under different scenarios with respect to the occurrence of intermediate events and measures of predictive accuracy. In Section 4, we present the results of the analyses of the two motivating studies, while in Section 5, we show the results of a simulation study. Finally, in Section 6, we close with a discussion.

## 2 JOINT MODEL FOR LONGITUDINAL AND TIME‐TO‐EVENT DATA WITH AN INTERMEDIATE EVENT

Assuming *n* individuals under study, let $D_{n}=\{T_{i},\delta_{i},y_{i},\rho_{i};i=1,\text{…},n\}$ denote the sample from the target population, where $T_{i}=\min(T_{i}^{*},C_{i})$ denotes the observed event time, which is defined as the minimum value between the true event time $T_{i}^{*}$ and the censoring time *C*_*i*_, and $\delta_{i}=I(T_{i}^{*}\leq C_{i})$ the event indicator with *I*(·) being the indicator function, which is equal to 1 if $T_{i}^{*}\leq C_{i}$ and 0 otherwise. Moreover, let *ρ*_*i*_ denote the time to the intermediate event with a corresponding indicator *R*_*i*_(*t*)=*I*(*t* ≥ *ρ*_*i*_) at any time *t* during follow‐up, which takes the value 1 if a subject experienced the intermediate event and 0 otherwise. Furthermore, let $t_{i+}=\max(0,t_{ij}-\rho_{i};j=1,\text{…},n_{i})$ denote the time relative to the occurrence of the intermediate event and ***y***_*i*_ be the vector of size *n*_*i*_×1 of repeated measurements for the *i*th subject, with element *y*_*ij*_ being the observed value of the longitudinal outcome at time point *t*_*ij*_,*j*=1,…,*n*_*i*_. We assume ***y***_*i*_ to be a contaminated with measurement error version of the true and unobserved value of the longitudinal outcome at any time *t*: ***y***_*i*_(*t*)=***η***_*i*_(*t*)+***ϵ***_*i*_(*t*) with ***η***_*i*_(*t*) denoting the true value of the longitudinal outcome at time *t* and measurement error $\epsilon_{i}(t)∼N(0,\sigma^{2}I_{n_{i}})$. The true level of the longitudinal outcome is then formulated as

(1) $$\eta_{i}(t)=\left\{\begin{array}{cc} x_{i}^{⊤}(t)\beta +z_{i}^{⊤}(t)b_{i},\; & 0<t<\rho_{i}\\ x_{i}^{⊤}(t)\beta +z_{i}^{⊤}(t)b_{i}+{\tilde{x}}_{i}^{⊤}(t)\tilde{\beta }+{\tilde{z}}_{i}^{⊤}(t){\tilde{b}}_{i},\; & t\geq \rho_{i}, \end{array}\right.$$

where $x_{i}^{⊤}(t)$ and $z_{i}^{⊤}(t)$ are design vectors for the fixed‐effects regression coefficients ***β*** and the random‐effects ***b*_*i*_**, respectively. Design vectors ${\tilde{x}}_{i}^{⊤}(t)$ and ${\tilde{z}}_{i}^{⊤}(t)$ include any function of the time‐varying covariates *R*_*i*_(*t*) and *t*_*i*+_, which describe the changes of the longitudinal trajectory after the occurrence of the intermediate event. These changes are then captured by the corresponding fixed‐effects regression coefficients $\tilde{\beta }$ allowing for subject‐specific variation via the random‐effects ${\tilde{b}}_{i}$. The random‐effects ***b***_*i*_ and $\tilde{b_{i}}$ are assumed to be normally distributed with mean zero and a *q*×*q* variance‐covariance matrix ***D***.

Depending on how the trajectory of the biomarker changes after the occurrence of the intermediate event, the specification of ${\tilde{x}}_{i}^{⊤}(t)$ and ${\tilde{z}}_{i}^{⊤}(t)$ may vary. Let $g\{R_{i}(t),t_{i+}\}={\tilde{x}}_{i}^{⊤}(t)\tilde{\beta }+{\tilde{z}}_{i}^{⊤}(t){\tilde{b}}_{i}$ denote the part of (1), which describes the changes in the longitudinal profile after the occurrence of the intermediate event. Then, in a setting as the one illustrated in Figure 1, for the pulmonary gradient data set, where the longitudinal trajectory is characterized by a seemingly linear evolution before and after the occurrence of the intermediate event, a steep drop at the occurrence of the intermediate event, and a potential change in the slope after the occurrence of the intermediate event, function *g*{*R*_*i*_(*t*),*t*_*i*+_} could be specified as $R_{i}(t)({\tilde{\beta }}_{1}+{\tilde{b}}_{i1})+t_{i+}({\tilde{\beta }}_{2}+{\tilde{b}}_{i2})$. That is, the steep drop at the occurrence of the intermediate event will be captured by $\left({\tilde{\beta }}_{1}+{\tilde{b}}_{i1}\right)$ and the change in the slope after the occurrence of the intermediate event will be captured by $\left({\tilde{\beta }}_{2}+{\tilde{b}}_{i2}\right)$. On the other hand, in the setting of the SPRINT data where the longitudinal profiles show a nonlinear evolution over time without steep sudden changes, function *g*{*R*_*i*_(*t*),*t*_*i*+_} could be specified as $\sum_{k=0}^{Q}({\tilde{\beta }}_{k}+{\tilde{b}}_{ik})B_{k}(t_{i+},k)$, where *B*_*k*_(*t*_*i*+_,***k***) denotes the *k*th basis function of a B‐spline with knots *k*_1_,…,*k*_*Q*_. In that case, the change in the shape of the nonlinear longitudinal trajectory after the occurrence of the intermediate event will be captured by $\sum_{k=1}^{Q}({\tilde{\beta }}_{k}+{\tilde{b}}_{ik})$ while there is no need to include *R*_*i*_(*t*). Generally, the functional form of *g*{*R*_*i*_(*t*),*t*_*i*+_} may vary, allowing for a broad range of mixed‐effects models that can capture various types of changes in the longitudinal profile after the occurrence of the intermediate event.

Let $H_{i}(t,\rho_{i})=[\eta_{i}\{s,\rho_{i}(s)\},0\leq s\leq t]$ denote the history of the longitudinal outcome up to time *t*. Note that, in the definition of the history of the longitudinal outcome, we explicitly indicate that the true underlying value of the longitudinal outcome is also a function of the time to the intermediate event *ρ*_*i*_, ie, to highlight that the subject‐specific trajectory, *η*_*i*_(*t*), differs from the occurrence of the intermediate event onwards. Then, the effects of the longitudinal outcome and the intermediate event, while adjusting for other covariates, on the risk for an event are quantified by utilizing a relative risk model of the form

(2) $$h_{i}\{t|H_{i}(t,\rho_{i}),w_{i}\}=\left\{\begin{array}{cc} h_{0}(t)\exp\left[\gamma^{⊤}w_{i}+f_{t<\rho_{i}}{\left\{H_{i}\left(t,\rho_{i}\right),b_{i}\right\}}^{⊤}\alpha \right],\; & 0<t<\rho_{i}\\ h_{0}(t)\exp\left[\gamma^{⊤}w_{i}+1\zeta +f_{t\geq \rho_{i}}{\left\{H_{i}\left(t,\rho_{i}\right),b_{i}\right\}}^{⊤}\alpha \right],\; & t\geq \rho_{i}, \end{array}\right.$$

where *h*_0_(*t*) is the baseline risk function and ***w***_*i*_ is a vector of baseline covariates with a corresponding vector of regression coefficients *γ*. The effect of the intermediate event on the risk is captured by the regression coefficient *ζ*, which quantifies the change in risk from the occurrence of the intermediate event onwards. Furthermore, the hazard of an event for patient *i* at any time *t* is associated with the subject‐specific trajectory, *η*_*i*_(*t*), through $f\{(H_{i}(t,\rho_{i}),b_{i}\}$, which is a function of the history of the longitudinal outcome up to time $H_{i}(t,\rho_{i})$ and/or the vector of subject‐specific effects ***b***_*i*_. Function $f_{\left(t,\rho_{i}\right)}\{(H_{i}(t,\rho_{i}),b_{i}\}$ determines the association structure between the longitudinal and the time‐to‐event processes, while the corresponding vector of regression coefficients ***α*** quantifies the magnitude of the association. Several functional forms for the specification of the association structure have been used in the literature, such as the current value, current slope, and the cumulative effect.^10^ The functional form of the association structure is an important feature of the joint model formulation, especially with regard to the accuracy of the dynamic predictions.^4^, ^11^, ^12^ Hence, to allow for more flexibility, we explicitly allow for the functional form of the association structure to differ before and after the occurrence of the intermediate event. In general, any functional form can be used for $f_{t\geq \rho_{i}}\{H_{i}(t,\rho_{i}),b_{i}\}$ and $f_{t<\rho_{i}}\{H_{i}(t,\rho_{i}),b_{i}\}$ including, of course, the case where the association structure remains the same and $f_{t\geq \rho_{i}}\{H_{i}(t,\rho_{i}),b_{i}\}=f_{t<\rho_{i}}\{H_{i}(t,\rho_{i}),b_{i}\}$.

The estimation of the parameters of the proposed joint model is achieved under the Bayesian framework using Markov chain Monte Carlo algorithms. For more details regarding Bayesian estimation of joint models, the reader may refer to other works.^13^, ^14^, ^15^

## 3 INDIVIDUALIZED DYNAMIC PREDICTIONS WITH TIME‐VARYING INTERMEDIATE EVENTS

### 3.1 Dynamic predictions

Based on the joint model fitted in the sample $D_{n}=\{T_{i},\delta_{i},y_{i},\rho_{i};i=1,\text{…},n\}$ from the target population, dynamic predictions for a new subject *j* from the same population can be derived up to a future time of interest *u*>*t* given his/her biomarker history $H_{j}(t)=[\eta_{j}\{s,R_{j}(s)\},0\leq s\leq t]$. Let $Y_{j}(t)=\{y_{j}\left(t_{jl}\right);0\leq t_{jl}\leq t,l=1,\text{…},n_{j}\}$ denote the history of observed biomarker values taken up to time *t* for patient *j*, and then under the Bayesian joint model framework, these predictions can be estimated using the corresponding posterior predictive distributions, namely,

(3) $$\begin{array}{ccc} \pi_{j}\left(u|t\right) & =Pr\left\{T_{j}^{*}\geq u|T_{j}^{*}>t,Y_{j}(t),\theta \right\} & \\ & =\int \frac{S_{j}\left\{u|H_{j}(u,b_{j}),\theta \right\}}{S_{j}\{t|H_{j}(t,b_{j}),\theta \}}p\left\{b_{j}|T_{j}^{*}>t,Y_{j}(t),\theta \right\}db_{j}\\ & =\int \exp\left[-\int_{t}^{u}h_{j}\{s|H_{j}(u,b_{j})\}ds\right]p\left\{b_{j}|T_{j}^{*}>t,Y_{j}(t),\theta \right\}db_{j},t\leq s\leq u. \end{array}$$

Note that, in Equation (3) and for the remainder of the text, covariates *w*_*j*_ and *x*_*j*_ are suppressed from notation for simplicity and without loss of generality. Expressing the fraction term in (3) as $\exp\{-\int_{t}^{u}h_{i}(s)ds\},t\leq s\leq u$ has two main advantages. First, it reduces the computational time required, since the denominator part $S_{j}\{t|H_{j}(t,b_{j}),\theta \}$ does not need to be computed anymore. Second, it improves the precision of numerical integration. The latter benefit is due to the fact that, by re‐expressing the fraction term in (3) as such, an adaptive Gauss‐Kronrod scheme can be deployed for the numerical computation of smaller regions of the target interval. This improves the precision of Gaussian quadrature since the quadrature points are spent for smaller regions of the interval.

Incorporating the time‐varying covariate *R*_*i*_(*t*) in both the longitudinal and relative risk submodels of the joint model allows us to evaluate how the occurrence of the intermediate event of interest at a future time point will influence the individualized risk predictions, for subjects who have not experienced the intermediate event by time *t*. The main difference of our approach when compared with the approach of Sène et al^7^ is that we assume that both the instantaneous risk of the primary endpoint and the longitudinal profile change after the occurrence of the intermediate event, whereas they assumed an extrapolated longitudinal profile instead. More specifically, by assuming an extrapolated longitudinal profile, Sène et al^7^ were more interested in assessing how predictions change with and without a second treatment, whereas we are more interested in studying how individualized risk predictions are influenced by intermediate events, such as reintervention or adverse events, by explicitly allowing for changes in both the longitudinal and survival submodels.

That is, different scenarios regarding the time of the intermediate event may lead to changes in the risk captured via different individual dynamic predictions accordingly. More specifically, for a future time of interest *u*>*t* different assumptions can be made. (1) The patient experiences an intermediate event immediately *ρ*_*i*_=*t* or at a time point within the time interval of prediction *t* ≤ *ρ*_*i*_ ≤ *u*. (2) The patient does not experience an intermediate event within the time interval of prediction *ρ*_*i*_>*u*. The individualized dynamic predictions in (4) are then further dependent on the scenario of choice

(4) $$\pi_{j}(u|t,\rho_{j})=\left\{\begin{array}{cc} \int \exp\left\{-\int_{t}^{u}h_{j}(s|b_{j},\rho_{j}\leq u)ds\right\}p\left\{b_{j}|T_{j}^{*}>t,Y_{j}(t,t\leq \rho_{j}\leq u),\theta \right\}db_{j},\; & t\leq \rho_{j}\leq u\\ \int \exp\left\{-\int_{t}^{u}h_{j}(s|b_{j},\rho_{j}>u)ds\right\}p\left\{b_{j}|T_{j}^{*}>t,Y_{j}(t,t\leq u\leq \rho_{j}),\theta \right\}db_{j},\; & t\leq u<\rho_{j}. \end{array}\right.$$

In Equation (4), the full conditional posterior density of the random effects can be analytically expressed as $\left\{p(T_{j}^{*}>t|\rho_{j}\leq u,b_{j})\}\prod_{l}\{p(y_{j}|\rho_{j}\leq u,b_{j})\}p(b_{j}|\theta \right)$ for *t* ≤ *ρ*_*j*_ ≤ *u* and as $\left\{p(T_{j}^{*}>t|\rho_{j}\leq u,b_{j})\}\prod_{l}\{p(y_{j}|\rho_{j}>u,b_{j})\}p(b_{j}|\theta \right)$ for *t* ≤ *u*<*ρ*_*j*_, where $\prod_{l}\{p(y_{j}|\rho_{j}\leq u,b_{j})\}$ and $\prod_{l}\{p(y_{j}|\rho_{j}>u,b_{j})\}$ are multivariate Gaussian joint densities for the longitudinal responses with means $x_{j}^{⊤}(t)\beta +R_{j}(t)\beta_{R}+t_{+}\beta_{t_{+}}+z_{j}^{⊤}(t)b_{j}+R_{j}(t)b_{jR}+t_{+}b_{jt_{+}}$ and $x_{j}^{⊤}(t)\beta +z_{j}^{⊤}(t)b_{j}$, respectively, and variance‐covariance matrix $\sigma^{2}I_{n_{j}}$. *p*(***b***_*j*_|***θ***) is a multivariate Gaussian density function with mean 0 and variance covariance matrix *D*.

To estimate *π*_*j*_(*u*|*t*,*ρ*_*j*_), a Monte Carlo scheme is employed, for the integration over the random effects, where a large set of *θ* ^*m*^(*m*=1,…,*M*) and $b_{j}^{m}(m=1,\text{…},M)$ are sampled from their posterior distributions and subsequently used to compute $\pi_{j}^{m}(u|t,\rho_{j})$. The median value of $\pi_{j}^{m}(u|t,\rho_{j})$ is the point estimate and the 2.5% and 97.5% percentiles give a 95% credible interval.

### 3.2 Evaluation of predictive performance

To assess the performance of the individualized dynamic predictions described in the previous section, we will work under a similar framework as the one presented in the work of Rizopoulos et al.^4^ More specifically, we will use the time‐dependent area under the receiver operating characteristic curve (AUC) and the expected prediction error (PE) adapted for the presence of intermediate events.

Under the framework presented in Sections 2 and 3.1, a rule can be defined using the individualized dynamic predictions *π*_*j*_(*u*=*t*+Δ*t*|*t*) while utilizing the available longitudinal measurements up to *t*, $Y_{j}(t)$. More specifically, a subject *j* can be termed as either to experience the event *π*_*j*_(*u*=*t*+Δ*t*|*t*) ≤ *c* or not *π*_*j*_(*u*=*t*+Δ*t*|*t*)>*c* within a clinically relevant time interval (*t*,Δ*t*], with *c*∈[0,1]. That is, for a pair of subjects which is randomly chosen {*i*,*j*} for both of which the measurements up to *t* are provided, the AUC, which is calculated for varying values of *c*, is a measure of the discriminative capability of the assumed model and is given by

(5) $$\text{AUC}(t,\Delta t)=\mathrm{Pr}\left[\pi_{i}(t+\Delta t|t)<\pi_{j}(t+\Delta t|t)|\left\{T_{i}^{*}\in (t,t+\Delta t]\right\}\cap \left\{T_{j}^{*}>t+\Delta t\right\}\right],$$

which intuitively means that we expect the assumed model to give higher probability of surviving longer than the time interval of interest (*t*+Δ*t*] to the subject who did not experience the event (in this case, subject *j*).

However, in the presence of intermediate events, the dynamic predictions change depending on whether a subject experienced the intermediate event or not. That is, the AUC in (5) changes to

$$\text{AUC}(t,\Delta t)=\left\{\begin{array}{cc} \mathrm{Pr}\left[\pi_{i}(t+\Delta t|t,\rho_{i}>t)<\pi_{j}(t+\Delta t|t,\rho_{i}>t)|\left\{T_{i}^{*}\in (t,t+\Delta t]\right\}\cap \left\{T_{j}^{*}>t+\Delta t\right\}\right],\; & 0<t<\rho_{i},\rho_{j}\\ \mathrm{Pr}\left[\pi_{i}\left(t+\Delta t|t,\rho_{i}\leq t\right)<\pi_{j}\left(t+\Delta t|t,\rho_{i}\leq t\right)|\left\{T_{i}^{*}\in \left(t,t+\Delta t\right]\right\}\cap \left\{T_{j}^{*}>t+\Delta t\right\}\right],\; & t\geq \rho_{i},\rho_{j}. \end{array}\right.$$

Estimation of AUC(*t*,Δ*t*) is based in counting the concordant pairs of subjects by appropriately distinguishing between the comparable and the noncomparable (due to censoring) pairs of subjects at time *t*. More specifically, the following decomposition is used:

$$\hat{\text{AUC}}(t,\Delta t)={\hat{\text{AUC}}}_{1}(t,\Delta t)+{\hat{\text{AUC}}}_{2}(t,\Delta t)+{\hat{\text{AUC}}}_{3}(t,\Delta t)+{\hat{\text{AUC}}}_{4}(t,\Delta t).$$

Term ${\hat{\text{AUC}}}_{1}(t,\Delta t)$ refers to the pairs of subjects who are comparable

$$\Omega_{ij}^{\left(1\right)}(t)=\left\{\begin{array}{c} \left[\left\{T_{i}\in \left(t,t+\Delta t\right]\}\cap \{\delta_{i}=1\}\cap \{0<t<\rho_{i}\right\}\right]\cap \left[\left\{T_{j}>t+\Delta t\}\cap \{0<t<\rho_{j}\right\}\right],0<t<\rho_{i},\rho_{j}\\ \left[\left\{T_{i}\in \left(t,t+\Delta t\right]\}\cap \{\delta_{i}=1\}\cap \{t\geq \rho_{i}\right\}\right]\cap \left[\left\{T_{j}>t+\Delta t\}\cap \{t\geq \rho_{j}\right\}\right],t\geq \rho_{i},\rho_{j}, \end{array}\right.$$

where *i*,*j*=1,…,*n* with *i*≠*j*. We can then estimate and compare the survival probabilities *π*_*i*_(*t*+Δ*t*|*t*,*t* ≥ *ρ*_*i*_) and *π*_*j*_(*t*+Δ*t*|*t*,*t* ≥ *ρ*_*j*_) for subjects *i* and *j* who did not experience the intermediate event and *π*_*i*_(*t*+Δ*t*|*t*,0<*t*<*ρ*_*i*_) and *π*_*j*_(*t*+Δ*t*|*t*,0<*t*<*ρ*_*j*_) for subjects who experienced the intermediate event. Then, ${\hat{\text{AUC}}}_{1}(t,\Delta t)$ is the proportion of concordant subjects out of the set of comparable subjects at time *t*

$$\begin{array}{ll} {\hat{\text{AUC}}}_{1}\left(t,\Delta t\right) & =\frac{\underset{i\in A}{\sum }\underset{j\neq i\in A}{\sum }I\left\{{\hat{\pi }}_{i}\left(t+\Delta t|t,t<\rho_{i}\right)<{\hat{\pi }}_{j}(t+\Delta t|t,t<\rho_{j})\right\}\times I\left\{\Omega_{ij}^{\left(1\right)}(t)\right\}}{\underset{i\in A}{\sum }\underset{j\neq i\in A}{\sum }I\left\{\Omega_{ij}^{\left(1\right)}(t)\right\}}\\ & \;+\frac{\underset{i\in B}{\sum }\underset{j\neq i\in B}{\sum }I\left\{{\hat{\pi }}_{i}\left(t+\Delta t|t,t\geq \rho_{i}\right)<{\hat{\pi }}_{j}(t+\Delta t|t,t\geq \rho_{j})\right\}\times I\{\Omega_{ij}^{\left(1\right)}(t)\}}{\underset{i\in B}{\sum }\underset{j\neq i\in B}{\sum }I\left\{\Omega_{ij}^{\left(1\right)}(t)\right\}}, \end{array}$$

where $A=\{i,j:t<\rho_{i};i,j=1,\text{…},n\}$ and $B=\{i,j:t\geq \rho_{i};i,j=1,\text{…},n\}$. The remaining terms, ${\hat{\text{AUC}}}_{2}(t,\Delta t)$, ${\hat{\text{AUC}}}_{3}(t,\Delta t)$, and ${\hat{\text{AUC}}}_{4}(t,\Delta t)$ refer to the pairs of subjects who due to censoring cannot be compared, namely,

$$\;\Omega_{ij}^{\left(2\right)}(t)=\left\{\begin{array}{c} \left[\left\{T_{i}\in \left(t,t+\Delta t\right]\}\cap \{\delta_{i}=0\}\cap \{0<t<\rho_{i}\right\}\right]\cap \left[\left\{T_{j}>t+\Delta t\}\cap \{0<t<\rho_{j}\right\}\right],0<t<\rho_{i},\rho_{j}\\ \left[\left\{T_{i}\in \left(t,t+\Delta t\right]\}\cap \{\delta_{i}=0\}\cap \{t\geq \rho_{i}\right\}\right]\cap \left[\left\{T_{j}>t+\Delta t\}\cap \{t\geq \rho_{j}\right\}\right],t\geq \rho_{i},\rho_{j}, \end{array}\right.$$

$$\Omega_{ij}^{\left(3\right)}(t)=\left\{\begin{array}{c} \left[\left\{T_{i}\in \left(t,t+\Delta t\right]\}\cap \{\delta_{i}=1\}\cap \{0<t<\rho_{i}\right\}\right]\cap \left[\left\{T_{i}<T_{j}\leq t+\Delta t\}\cap \{0<t<\rho_{j}\}\cap \{\delta_{j}=0\right\}\right],0<t<\rho_{i},\rho_{j}\\ \left[\left\{T_{i}\in \left(t,t+\Delta t\right]\}\cap \{\delta_{i}=1\}\cap \{t\geq \rho_{i}\right\}\right]\cap \left[\left\{T_{i}<T_{j}\leq t+\Delta t\}\cap \{t\geq \rho_{j}\}\cap \{\delta_{j}=0\right\}\right],t\geq \rho_{i},\rho_{j}, \end{array}\right.$$

$$\Omega_{ij}^{\left(4\right)}(t)=\left\{\begin{array}{c} \left[\left\{T_{i}\in \left(t,t+\Delta t\right]\}\cap \{\delta_{i}=0\}\cap \{0<t<\rho_{i}\right\}\right]\cap \left[\left\{T_{i}<T_{j}\leq t+\Delta t\}\cap \{0<t<\rho_{j}\}\cap \{\delta_{j}=0\right\}\right],0<t<\rho_{i},\rho_{j}\\ \left[\left\{T_{i}\in \left(t,t+\Delta t\right]\}\cap \{\delta_{i}=0\}\cap \{t\geq \rho_{i}\right\}\right]\cap \left[\left\{T_{i}<T_{j}\leq t+\Delta t\}\cap \{t\geq \rho_{j}\}\cap \{\delta_{j}=0\right\}\right],t\geq \rho_{i},\rho_{j}, \end{array}\right.$$

which contribute to the overall AUC after being appropriately weighted with the probability that they would be comparable

$$\begin{array}{ll} {\hat{\text{AUC}}}_{m}\left(t,\Delta t\right) & =\frac{\underset{i\in A}{\sum }\underset{j\neq i\in A}{\sum }I\left\{{\hat{\pi }}_{i}\left(t+\Delta t|t,t<\rho_{i}\right)<{\hat{\pi }}_{j}(t+\Delta t|t,t<\rho_{j})\right\}\times I\left\{\Omega_{ij}^{\left(m\right)}(t)\right\}\times {\hat{\nu }}_{ij}^{\left(m\right)}}{\underset{i\in A}{\sum }\underset{j\neq i\in A}{\sum }I\left\{\Omega_{ij}^{\left(m\right)}(t)\right\}\times \hat{\nu_{ij}^{\left(m\right)}}}\\ & \;+\frac{\underset{i\in B}{\sum }\underset{j\neq i\in B}{\sum }I\left\{{\hat{\pi }}_{i}(t+\Delta t|t,t\geq \rho_{i})<{\hat{\pi }}_{j}(t+\Delta t|t,t\geq \rho_{j})\right\}\times I\left\{\Omega_{ij}^{\left(m\right)}(t)\right\}\times {\hat{\nu }}_{ij}^{\left(m\right)}}{\underset{i\in B}{\sum }\underset{j\neq i\in B}{\sum }I\left\{\Omega_{ij}^{\left(m\right)}(t)\right\}\times {\hat{\nu }}_{ij}^{\left(m\right)}}, \end{array}$$

with *m*=2,3,4 and

$$\begin{array}{ll} {\hat{\nu }}_{ij}^{\left(2\right)} & =\left\{\begin{array}{cc} 1-{\hat{\pi }}_{i}\left(t+\Delta t|T_{i},t<\rho_{i}\right),\; & i\in A\\ 1-{\hat{\pi }}_{i}\left(t+\Delta t|T_{i},t\geq \rho_{i}\right),\; & i\in B, \end{array}\right.\\ {\hat{\nu }}_{ij}^{\left(3\right)} & =\left\{\begin{array}{cc} {\hat{\pi }}_{j}(t+\Delta t|T_{j},t<\rho_{j}),\; & j\in A\\ {\hat{\pi }}_{j}(t+\Delta t|T_{j},t\geq \rho_{j}),\; & j\in B, \end{array}\right.\\ {\hat{\nu }}_{ij}^{\left(4\right)} & =\left\{\begin{array}{cc} \{1-{\hat{\pi }}_{i}(t+\Delta t|T_{i},t<\rho_{i})\}\times {\hat{\pi }}_{j}(t+\Delta t|T_{j},t<\rho_{j}),\; & i,j\in A\\ \{1-{\hat{\pi }}_{i}(t+\Delta t|T_{i},t\geq \rho_{i})\}\times {\hat{\pi }}_{j}(t+\Delta t|T_{j},t\geq \rho_{j}),\; & i,j\in B. \end{array}\right. \end{array}$$

The expected error of predicting future events can be used to assess the accuracy of individualized dynamic predictions. Similarly, as for the AUC, to account for the dynamic nature of the longitudinal outcome, we focus our interest in predicting events that occur at a time point *u*>*t* given the information available up to time *t*, $Y_{j}(t)$. Let $N_{j}(t)=I(T_{i}^{*}>t)$ denote the event status of subject *j* at time *t*. Using the square loss function, the expected PE is then

(6) $$\text{PE}(u|t)=E\left[{\left\{N_{j}(u)-\pi_{j}(u|t)\right\}}^{2}\right],$$

where the expectation is taken with respect to the distribution of the event times. Adapting the above to the framework of intermediate events, (6) can be re‐expressed as

$$\text{PE}(u|t)=\left\{\begin{array}{cc} E\left[{\left\{N_{j}(u)-\pi_{j}(u|t,\rho_{i}>t)\right\}}^{2}\right],\; & 0<t<\rho_{i}\\ E\left[{\left\{N_{j}(u)-\pi_{j}(u|t,\rho_{i}\leq t)\right\}}^{2}\right],\; & t\geq \rho_{i}, \end{array}\right.$$

where, for each case, the corresponding individualized dynamic predictions showed in (4) are used. The estimate of PE(*u*|*t*) as proposed by Henderson et al^16^ and adjusted for the presence of intermediate events is given by

$$\begin{array}{ll} \hat{\text{PE}}\left(u|t,\rho_{i}\right) & ={\left\{n\left(t,t<\rho_{i}\right)\right\}}^{-1}\underset{i\in A,T_{i}\geq t}{\sum }I\left(T_{i}\geq u\right){\left\{1-{\hat{\pi }}_{i}\left(u|t,t<\rho_{i}\right)\right\}}^{2}+\delta_{i}I\left(T_{i}<u\right){\left\{0-{\hat{\pi }}_{i}\left(u|t,t<\rho_{i}\right)\right\}}^{2}\\ & \;+\left(1-\delta_{i}\right)I\left(T_{i}<u\right)\left[{\hat{\pi }}_{i}\left(u|T_{i},t<\rho_{i}\right){\left\{1-{\hat{\pi }}_{i}\left(u|t,t<\rho_{i}\right)\right\}}^{2}\{1-{\hat{\pi }}_{i}\left(u|T_{i},t<\rho_{i}\right)\}\right.\\ & \;\times \left.{\left\{0-{\hat{\pi }}_{i}\left(u|t,t<\rho_{i}\right)\right\}}^{2}\right]+{\left\{n\left(t,t\geq \rho_{i}\right)\right\}}^{-1}\underset{i\in B,T_{i}\geq t}{\sum }I\left(T_{i}\geq u\right){\left\{1-{\hat{\pi }}_{i}\left(u|t,t\geq \rho_{i}\right)\right\}}^{2}\\ & \;+\delta_{i}I\left(T_{i}<u\right){\left\{0-{\hat{\pi }}_{i}\left(u|t,t\geq \rho_{i}\right)\right\}}^{2}\left(1-\delta_{i}\right)I\left(T_{i}<u\right)\left[{\hat{\pi }}_{i}\left(u|T_{i},t\geq \rho_{i}\right){\left\{1-{\hat{\pi }}_{i}\left(u|t,t\geq \rho_{i}\right)\right\}}^{2}\right.\\ & \;\times \left.\{1-{\hat{\pi }}_{i}\left(u|T_{i},t\geq \rho_{i}\right)\}{\left\{0-{\hat{\pi }}_{i}\left(u|t,t<\rho_{i}\right)\right\}}^{2}\right], \end{array}$$

where *n*(*t*,*t*<*ρ*_*i*_) and *n*(*t*,*t* ≥ *ρ*_*i*_) denote the number of subjects still at risk at time *t*, who have not/have experienced the intermediate event, respectively.

## 4 ANALYSIS OF PULMONARY GRADIENT AND SPRINT TRIAL DATA

### 4.1 Pulmonary gradient data set

The pulmonary gradient data set was introduced in Section 1. Our goal is to investigate the association between the pulmonary gradient and the risk of death, how reoperation as an intermediate event changes the evolution of the pulmonary gradient and the instantaneous risk for death, and then to utilize this information to derive individualized dynamic predictions under different scenarios with respect to a future time of reoperation.

In Figure 1, the evolutions of pulmonary gradient for reoperated and non reoperated patients are depicted, where it is shown that for the case of reoperated patients the profiles are characterized by a linear increasing trend, which drops at the moment of reoperation and then continues to increase, whereas for the case of nonreoperated patients, the profiles show a linear increasing trend. Therefore, for this outcome, we assumed a linear mixed‐effects submodel including a linear effect of time, a drop at the moment of reoperation, and a change in slope after the occurrence of reoperation in both the fixed‐effects parts and random‐effects parts of the model while correcting for baseline differences in age and sex. A preliminary analysis suggested that assuming nonlinear effects of time did not improve the fit of the model to the data. Hence, we used the following specification for the mixed‐effects model:

$$PG_{i}(t)=\left\{\begin{array}{cc} (\beta_{0}+b_{i0})+(\beta_{1}+b_{i1})\times t_{i}+\beta_{4}Age_{i}+\beta_{5}Sex_{i}+\epsilon_{i}(t),\; & 0<t<\rho_{i}\\ (\beta_{0}+b_{i0})+(\beta_{1}+b_{i1})\times t_{i}+({\tilde{\beta }}_{2}+{\tilde{b}}_{i2})\times R_{i}(t)+({\tilde{\beta }}_{3}+{\tilde{b}}_{i3})\times t_{i+}+\beta_{4}\mathrm{Ag}e_{i}+\beta_{5}\mathrm{Se}x_{i}+\epsilon_{i}(t),\; & t\geq \rho_{i}, \end{array}\right.$$

where *PG*_*i*_(*t*) are the measurements of pulmonary gradient, *R*_*i*_(*t*) is a binary time‐dependent indicator of reoperation, and $t_{i+}=\max(0,t_{ij}-\rho_{ij};j=1,\text{…},n_{i})$ is the time relative to reoperation.

To investigate the association between the pulmonary gradient and the risk of death, we postulated relative risk submodels with different parametrizations for the pulmonary gradient. The baseline hazard was expressed as a B‐splines function. We also corrected for age and sex and assumed reoperation to have a direct effect on the hazard. Based on a preliminary analysis, we assessed various formulations for the association structure. However, only functional forms including the slope of the pulmonary gradient were found to be stronger. Assuming a different functional form after the occurrence of reoperation did not improve the fit of the model to the data. Therefore, we present the joint models that include the slope of the pulmonary gradient along with the joint model that assumes an association with the current value of the pulmonary gradient for the sake of comparison, since it is the most common form of the association structure

$$\begin{array}{ll} M1:h_{i}(t) & =h_{0}(t)\exp\{\gamma_{1}{\text{Age}}_{i}+\gamma_{2}{\text{Sex}}_{i}+\zeta R_{i}(t)+\alpha_{1}\eta_{PGi}(t)\},\\ M2:h_{i}(t) & =h_{0}(t)\exp\left\{\gamma_{1}{\text{Age}}_{i}+\gamma_{2}{\text{Sex}}_{i}+\zeta R_{i}(t)+\alpha_{1}\eta_{PGi}(t)+\alpha_{2}\frac{d}{dt}\eta_{PGi}(t)\right\},\\ M3:h_{i}(t) & =h_{0}(t)\exp\left\{\gamma_{1}{\text{Age}}_{i}+\gamma_{2}{\text{Sex}}_{i}+\zeta R_{i}(t)+\alpha_{2}\frac{d}{dt}\eta_{PGi}(t)\right\}. \end{array}$$

Table 1 summarizes the parameter estimates and the 95% credibility intervals of the longitudinal submodel that was used for the pulmonary gradient data set. Table 2 summarizes the parameter estimates and the 95% credibility intervals of the survival submodels based on the three joint models fitted to the pulmonary gradient data set. As shown in Table 2, the association of the pulmonary gradient with the instantaneous risk of death was weak regardless the functional form of the association structure. The strongest association in magnitude was found when using the slope parametrization both before and after the occurrence of the intermediate event.

**Table 1:** Estimated coefficients and 95% credibility intervals for the parameters of the longitudinal submodel fitted to the pulmonary gradient data set

	Est.	**95** *%* CI
*β*_0_	23.41	(20.677; 26.068)
*β*_1_	0.99	(0.797; 1.178)
${\tilde{\beta }}_{2}$	‐12.83	(‐18.735; ‐6.647)
${\tilde{\beta }}_{3}$	‐0.02	(‐0.994; 1.198)
*β*_4_	‐0.13	(‐0.226; ‐0.033)
*β*_5_	‐4.01	(‐6.671; ‐1.306)
*σ*	10.52	(10.262; 10.8)

**Table 2:** Estimated hazard ratios and 95% credibility intervals for the parameters of the survival submodels based on the three joint models fitted to the pulmonary gradient data set. Age at baseline is measured in years; *α*_1_ and *α*_2_ are measured in the units of the pulmonary gradient (mmHg) when referring to the current value association and in (mmHg/time) when referring to the current slope association

	Value		Value + Slope		Slope	
	HR	**95** *%* CI	HR	**95** *%* CI	HR	**95** *%* CI
*γ*_1_	1.05	(1.03; 1.078)	1.06	(1.03; 1.083)	1.06	(1.03; 1.08)
*γ*_2_	0.51	(0.223; 1.073)	0.52	(0.235; 1.099)	0.51	(0.25; 1.061)
*ζ*	0.34	(0.056; 1.399)	0.32	(0.042; 1.406)	0.31	(0.042; 1.255)
*α*_1_	1.01	(0.992; 1.031)	1.01	(0.983; 1.032)	1.32	(0.783; 1.919)
*α*_2_			1.17	(0.624; 1.987)		

**Figure 3:**
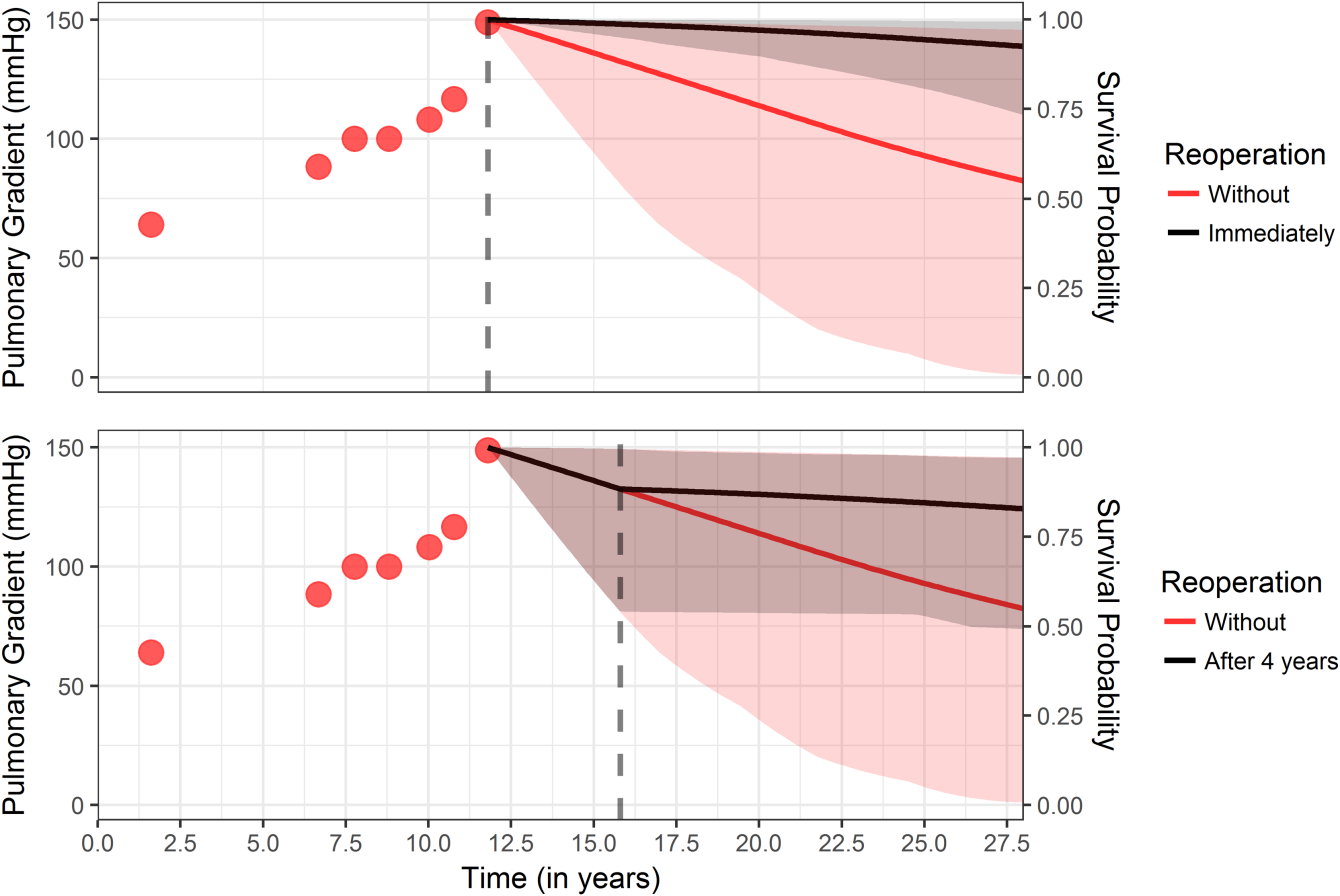
Survival probabilities for a new subject under different scenarios with respect to the timing of reoperation. The vertical gray dashed line depicts the time of reoperation [Colour figure can be viewed at http://wileyonlinelibrary.com]

Despite the weak magnitude of the association, reoperation was found to have a strong effect on the longitudinal evolution of pulmonary gradient. The fitted joint models can be used to show how individualized dynamic predictions can be derived for a new subject, under different scenarios with respect to the timing of reoperation in the future. For this illustration, we will use model M3 since the slope parametrization was found to have a stronger effect on the instantaneous risk of death. In Figure 3, the individual prediction of survival for a subject with seven measurements of pulmonary gradient, so far, is shown under the assumptions of no reoperation in the future versus reoperation immediately and after four, years respectively. As shown in Figure 3, reoperation improves the survival probability for the new subject regardless its timing. When the reoperation is not assumed to be immediate, the survival curves overlap up to the point of reoperation and then separate, depicting the improvement on the survival probability for this subject due to reoperation.

### 4.2 SPRINT data set

The SPRINT data set was also introduced in Section 1. Our goal is to investigate how SAEs during follow‐up change the evolution of the systolic blood pressure and the instantaneous risk for the composite endpoint, and then to utilize this information to derive individualized dynamic predictions under different scenarios with respect to the occurrence of SAEs in the future.

In Figure 2, a random sample of the evolutions of systolic blood pressure for patients who experienced and who did not experience SAEs is depicted. For both sets of patients, the profiles show diverse nonlinear trends, which we assume to change after the occurrence of SAEs. Therefore, for this outcome, we assumed a nonlinear mixed‐effects submodel using natural cubic splines with three knots for the effect of time, and the effect of time relative to the occurrence of SAEs in both the fixed‐effects parts and random‐effects parts of the model while adjusting for differences in treatment. More specifically, the following specification for the mixed‐effects model was used:

$$\mathrm{SB}P_{i}(t)=\left\{\begin{array}{cc} (\beta_{0}+b_{i0})+\left(\sum_{k=0}^{3}(\beta_{\left(k+1\right)}+b_{i(k+1)})B_{k}(t,k)\right)+\beta_{5}{\text{Treatment}}_{i}+\left(\sum_{k=0}^{3}\beta_{\left(k+6\right)}B_{k}(t,k)\right)\\ \times \;{\text{Treatment}}_{i}+\epsilon_{i}(t),\; & 0<t<\rho_{i}\\ (\beta_{0}+b_{i0})+\left(\sum_{k=0}^{3}(\beta_{\left(k+1\right)}+b_{i(k+1)})B_{k}(t,k)\right)+\beta_{5}{\text{Treatment}}_{i}+\left(\sum_{k=0}^{3}\beta_{\left(k+6\right)}B_{k}(t,k)\right)\\ \times \;{\text{Treatment}}_{i}+\left(\sum_{k=0}^{3}({\tilde{\beta }}_{\left(k+10\right)}+{\tilde{b}}_{i(k+10)})B_{k}(t_{+},k)\right)+\left(\sum_{k=0}^{3}{\tilde{\beta }}_{\left(k+14\right)}B_{k}(t_{+},k)\right)\\ \times \;{\text{Treatment}}_{i}+\epsilon_{i}(t),\; & t\geq \rho_{i}, \end{array}\right.$$

where SBP_*i*_(*t*) are the measurements of systolic blood pressure and $t_{i+}=\max(0,t_{ij}-\rho_{ij};j=1,\text{…},n_{i})$ is the time relative to the occurrence of SAE.

To investigate the association between the systolic blood pressure and the composite endpoint, we postulated relative risk submodels with different parametrizations for the systolic blood pressure. The baseline hazard was expressed as a B‐splines function. We also corrected for treatment and assumed the occurrence of SAE to have a direct effect on the hazard. The functional forms we used for the association structure were the current value, slope, area, both the current value and slope, as well as more elaborate ones assuming that the value, slope, and area all have an effect on the hazard and assuming that after the occurrence of the SAE the effect of the current slope on the hazard changes. The joint models are given in detail as follows:

$$\begin{array}{ll} M4:h_{i}(t) & =h_{0}(t)\exp\{\gamma_{1}{\text{Treatment}}_{i}+\zeta R_{i}(t)+\alpha_{1}\eta_{\mathrm{SBP}i}(t)\},\\ M5:h_{i}(t) & =h_{0}(t)\exp\left\{\gamma_{1}{\text{Treatment}}_{i}+\zeta R_{i}(t)+\alpha_{2}\frac{d}{dt}\eta_{\mathrm{SBP}i}(t)\right\},\\ M6:h_{i}(t) & =h_{0}(t)\exp\left\{\gamma_{1}{\text{Treatment}}_{i}+\zeta R_{i}(t)+\alpha_{1}\eta_{\mathrm{SBP}i}(t)+\alpha_{2}\frac{d}{dt}\eta_{\text{SBP}i}(t)\right\}.\\ M7:h_{i}(t) & =h_{0}(t)\exp\left\{\gamma_{1}{\text{Treatment}}_{i}+\zeta R_{i}(t)+\alpha_{3}\int_{0}^{t}\eta_{\mathrm{SBP}i}(s)ds\right\}.\\ M8:h_{i}(t) & =h_{0}(t)\exp\left\{\gamma_{1}{\text{Treatment}}_{i}+\zeta R_{i}(t)+\alpha_{1}\eta_{\mathrm{SBP}i}(t)+\alpha_{2}\frac{d}{dt}\eta_{\mathrm{SBP}i}(t)+\alpha_{3}\int_{0}^{t}\eta_{\mathrm{SBP}i}(s)ds\right\}.\\ M9:h_{i}(t) & =h_{0}(t)\exp\left\{\gamma_{1}{\text{Treatment}}_{i}+\zeta R_{i}(t)+\alpha_{1}\eta_{\mathrm{SBP}i}(t)+\alpha_{2}\frac{d}{dt}\eta_{\mathrm{SBP}i}(t)+\alpha_{3}R_{i}(t)\times \frac{d}{dt}\eta_{\mathrm{SBP}i}(t)\right\}. \end{array}$$

Table 3 summarizes the parameter estimates and the 95% credibility intervals of the longitudinal submodel that were used for the SPRINT data set. Table 4 summarizes the parameter estimates and the 95% credibility intervals of the survival submodels based on the six joint models fitted to the SPRINT data set. As shown in Table 4, the association of the pulmonary gradient with the instantaneous risk of composite endpoint was weak in magnitude but significant in the cases of value and slope association and area association.

**Table 3:** Estimated coefficients and 95% credibility intervals for the parameters of the longitudinal submodel fitted to the SPRINT data set

	Est.	**95** *%* CI
*β*_0_	137.95	(137.549; 138.32)
*β*_1_	‐1.33	(‐1.808; ‐0.851)
*β*_2_	‐0.40	(‐0.873; 0.05)
*β*_3_	‐8.39	(‐9.302; ‐7.505)
*β*_4_	1.01	(0.574; 1.407)
*β*_5_	‐0.57	(‐1.116; 0.021)
*β*_6_	‐13.45	(‐14.125; ‐12.778)
*β*_7_	‐11.10	(‐11.734; ‐10.456)
*β*_8_	‐25.93	(‐27.184; ‐24.583)
*β*_9_	‐9.51	(‐10.094; ‐8.938)
${\tilde{\beta }}_{10}$	0.06	(‐0.954; 1.088)
${\tilde{\beta }}_{11}$	‐0.06	(‐1.282; 1.181)
${\tilde{\beta }}_{12}$	‐1.82	(‐3.097; ‐0.389)
${\tilde{\beta }}_{13}$	‐0.62	(‐2.402; 1.268)
${\tilde{\beta }}_{14}$	1.84	(0.423; 3.333)
${\tilde{\beta }}_{15}$	0.35	(‐1.321; 2.107)
${\tilde{\beta }}_{16}$	5.06	(2.983; 6.847)
${\tilde{\beta }}_{17}$	1.48	(‐1.028; 3.808)
*σ*	11.22	(11.164; 11.266)

**Table 4:** Estimated hazard ratios and 95% credibility intervals for the parameters of the joint models fitted to the SPRINT data set. *α*_1_, *α*_2_, and *α*_3_ are measured in the units of 20 times systolic blood pressure (20mmHg) when referring to the current value association, in (10mmHg/time) when referring to the current slope association, and in (20mmHg×*t*) when referring to the area under the curve association

	Value		Slope		Value + Slope		Area		Value + Slope + Area		Value + Slope Int	
	HR	**95** *%* CI	HR	**95** *%* CI	HR	**95** *%* CI	HR	**95** *%* CI	HR	**95** *%* CI	HR	**95** *%* CI
*γ*_1_	0.91	(0.59; 0.859)	0.72	(0.59; 0.859)	0.9	(0.743; 1.093)	0.81	(0.69; 0.97)	0.9	(0.768; 1.054)	0.9	(0.737; 1.116)
*ζ*	1.86	(1.592; 2.379)	1.93	(1.592; 2.379)	1.89	(1.536; 2.294)	1.88	(1.567; 2.236)	1.87	(1.649; 2.154)	1.58	(1.203; 2.065)
*α*_1_	1.34	(0.940; 1.310)	1.11	(0.940; 1.310)	1.390	(1.166; 1.646)	1.07	(1.000; 1.143)	1.32	(1.173; 1.471)	1.37	(1.118; 1.671)
*α*_2_					1.100	(1.012; 1.206)			1.09	(1.049; 1.130)	1.05	(0.964; 1.158)
*α*_3_									1.02	(0.985; 1.059)	1.84	(0.985; 2.571)

**Figure 4:**
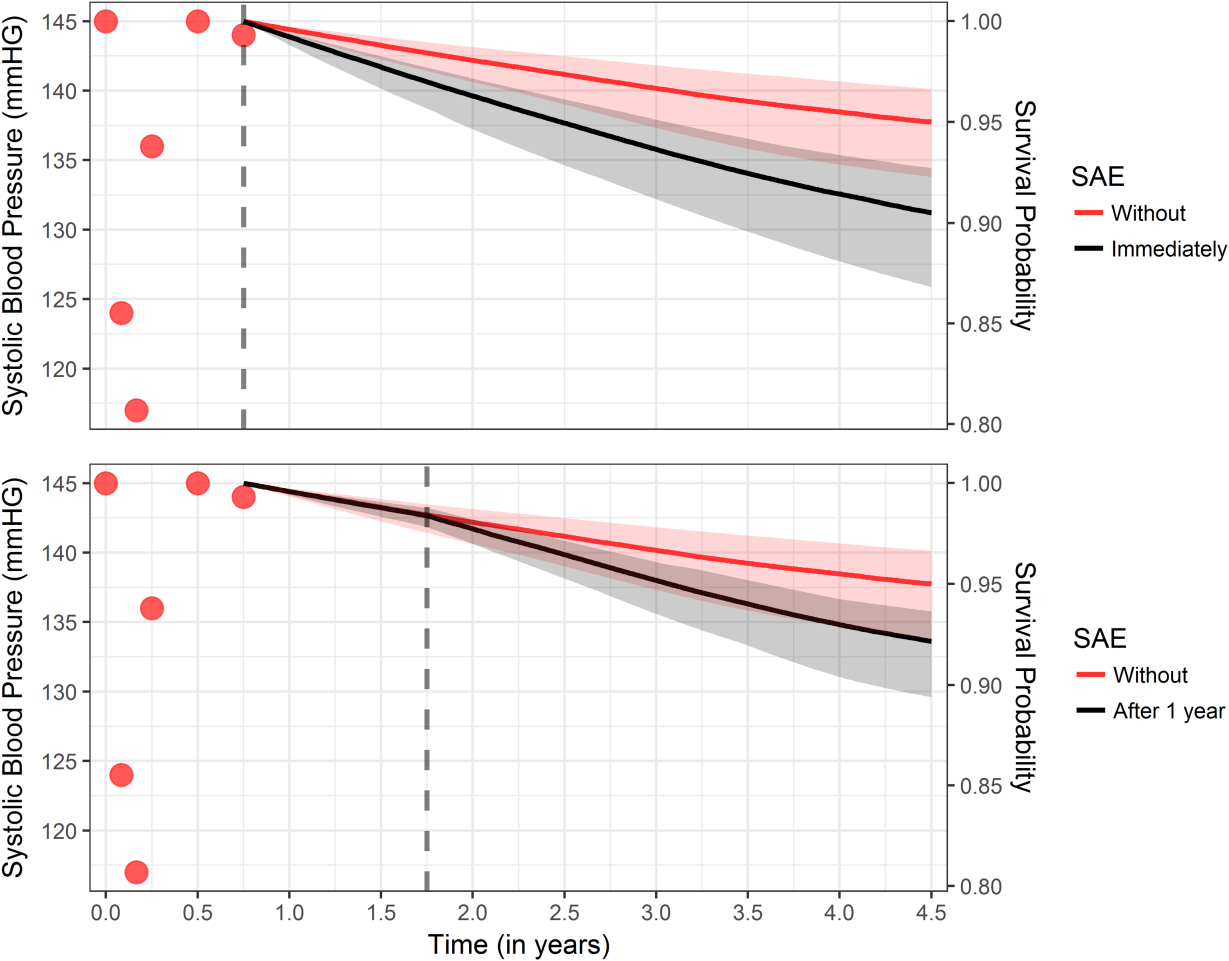
Survival probabilities for a new subject under different scenarios with respect to the occurrence of serious adverse event (SAE). The vertical gray dashed line depicts the time of SAE [Colour figure can be viewed at http://wileyonlinelibrary.com]

Similarly, as for the pulmonary gradient data set, in Figure 4, we illustrate how the individualized subject‐specific predictions for the composite endpoint of interest change under different scenarios for the timing of the occurrence of a SAE. More specifically, we illustrate the cases of an immediate occurrence of the adverse event and an occurrence after a year using the joint model that postulates effects of both the current value and current slope of the systolic blood pressure trajectory on the instantaneous risk of the composite endpoint. In both cases, the occurrence of the SAE worsens the survival probability considerably.

Finally, as an illustration of the use of the predictive performance measures, as they were presented in Section 3, we refer the reader to the online Supplementary Material S2 where we present a comparison in terms of the AUC and the PE for the SPRINT data where the extrapolation method (that is ignoring the longitudinal data observed after the occurrence of the intermediate event) and the proposed model are compared.

### 4.3 Software

The R package **JMbayes** was extended to appropriately account for the occurrence of intermediate events and deriving individualized dynamic predictions under different scenarios with respect to the occurrence of the intermediate event. These changes are already integrated in the package both on CRAN and in the development version on GitHub. However, since the specification of such models can be very application‐specific in terms of special features, which need to be accounted for due to the occurrence of the intermediate event, the data sets need to be appropriately prepared before their use with the functions of package **JMbayes**. The reader may refer to the online Supplementary Material Section S3 for a step‐by‐step tutorial on how to fit joint models with the occurrence of intermediate events and derive predictions thereafter. Finally, all the analyses presented in Section 4 were performed using R version 3.5.1 and package **JMbayes**.

## 5 SIMULATION STUDY

### 5.1 Design

To evaluate the performance of the proposed models and to compare, in terms of predictive accuracy, the dynamic predictions that account for the whole biomarker trajectory against the cases where extrapolation or a simple time‐dependent Cox model without accounting for the longitudinal data are assumed, we performed a simulation study. The main goal of the simulation study is to show the benefit in the accuracy of the individualized dynamic predictions when assuming that the intermediate event changes both the risk for the event of interest and the longitudinal trajectory against the case of assuming that the intermediate event only changes the risk for the event of interest while the longitudinal trajectory is extrapolated and the case where the longitudinal data are not taken into account. We assumed 2000 patients and then randomly selected follow‐up visits. Each visit time *t*_*ij*_ was simulated from a uniform distribution between 0 and 30. We assumed a total number of 20 measurements per subject. The final number of measurements, however, varies depending on when a subject experienced the clinical event or was censored. To mimic a realistic situation, the timing of the intermediate event was assumed to depend on the value of the biomarker trajectory. Specifically, if the biomarker exceeded a specific value, then reintervention took place at the next visit. For the cases that this value was not reached, the patient was assumed to never have experienced the intermediate event. For simplicity, we assumed a linear mixed‐effects model and a survival submodel without any baseline covariates.

For the continuous longitudinal outcome, we simulated the data from a linear mixed‐effects models similar to the model that we used for the pulmonary gradient data set

$$y_{i}(t)=\eta_{i}(t)+\epsilon_{i}(t)=(\beta_{0}+b_{i0})+(\beta_{1}+b_{i1})t_{i}+({\tilde{\beta }}_{2}+{\tilde{b}}_{i2})R_{i}(t)+({\tilde{\beta }}_{3}+{\tilde{b}}_{i3})t_{i+}+\epsilon_{i}(t),$$

where $\epsilon_{i}(t)∼N(0,\sigma^{2}I_{n_{i}})$ and $b_{i}={\left(b_{i0},b_{i1},{\tilde{b}}_{i2},{\tilde{b}}_{i3}\right)}^{⊤}∼N(0,D)$. More specifically, we adopted a linear effect for time, a “drop” effect that occurs at the time of reoperation and an effect for the change in the slope from the time of reoperation onwards for both the fixed and the random part. Time *t* was simulated from a uniform distribution between 0 and 30. Based on this model for the continuous outcome, we assumed three different scenarios

- $\text{Scenario 1:}\beta_{1}=20.7,\beta_{2}=1.6,{\tilde{\beta }}_{3}=-15.5,{\tilde{\beta }}_{4}=-0.76$,
- $\text{Scenario 2:}\beta_{1}=20.7,\beta_{2}=1.6,{\tilde{\beta }}_{3}=-15.5,{\tilde{\beta }}_{4}=0$,
- $\text{Scenario 3:}\beta_{1}=20.7,\beta_{2}=1.6,{\tilde{\beta }}_{3}=0,{\tilde{\beta }}_{4}=-0.76$.

**Figure 5:**
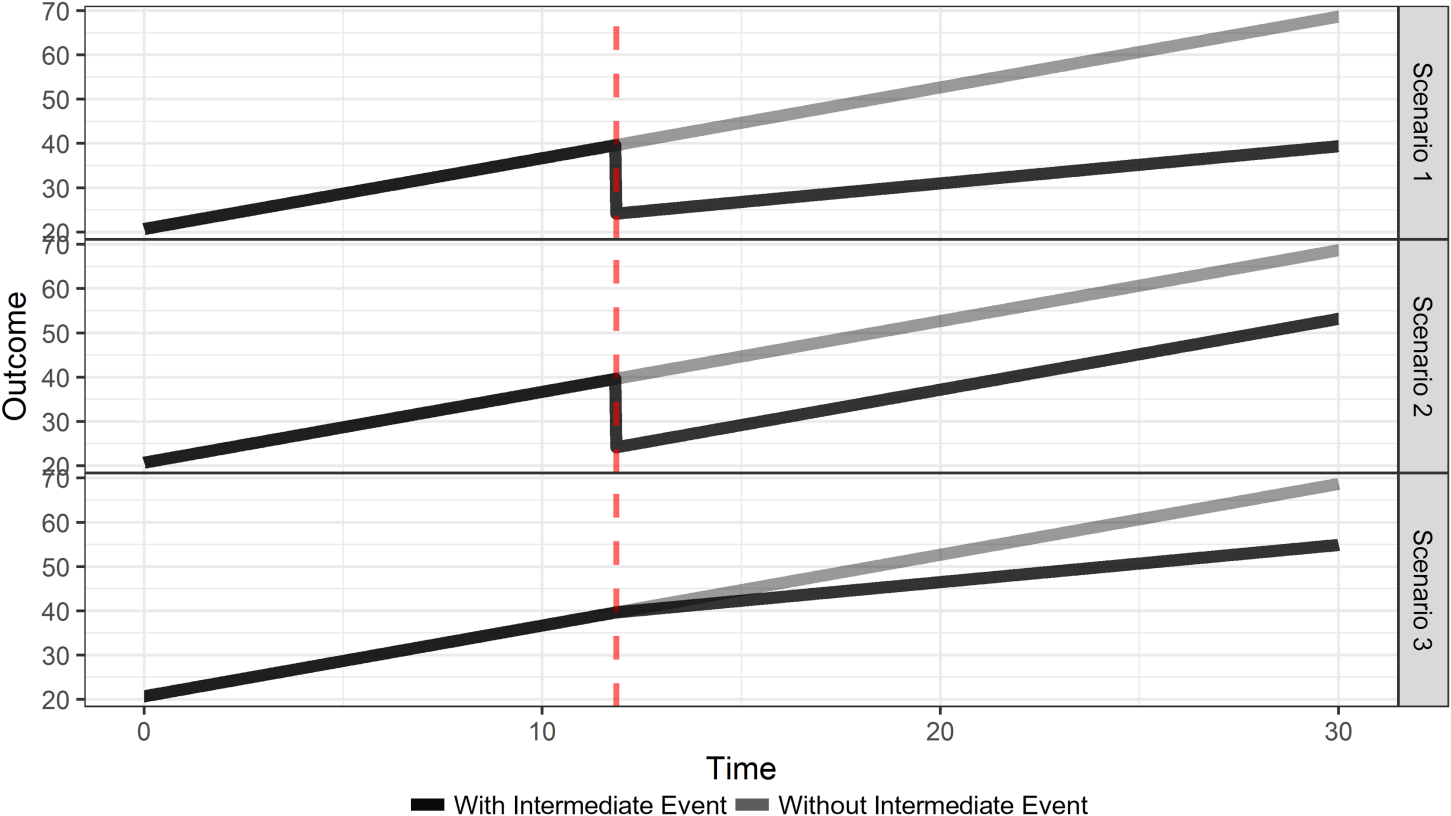
Assumed average longitudinal evolutions under the three simulation scenarios for subjects who experienced an intermediate event and for subjects who did not. The vertical red dashed line depicts the occurrence of the intermediate event [Colour figure can be viewed at http://wileyonlinelibrary.com]

The assumed longitudinal trajectories for each of the three scenarios are depicted in Figure 5.

More specifically, in the first scenario, we assume that the longitudinal profile drops at the occurrence of the intermediate, while the slope changes after its occurrence. In the second and third scenarios, the slope does not change after the occurrence of the intermediate event and the longitudinal profile does not drop, respectively.

For the survival outcome, we assumed a relative risk model of the form

$$h_{i}(t)=h_{0}(t)\exp\{\gamma 1+R_{i}(t)\zeta +\alpha_{1}\eta_{i}(t)\},$$

where **1** is a vector of ones for the intercept term with a corresponding regression coefficient *γ*, while the baseline risk was simulated from a Weibull distribution *h*_0_(*t*)=*ξt* ^*ξ*−1^ with *ξ*=20.4. The censoring process was assumed to follow an exponential distribution with mean equal to 22.6. For the time‐dependent Cox model, the following relative risk model was used instead:

$$h_{i}(t)=h_{0}(t)\exp\{\gamma 1+R_{i}(t)\zeta \},$$

where no association with the longitudinal outcome is assumed.

### 5.2 Results

Under the settings described in the previous section, 500 data sets were simulated for each of the three scenarios. All the data sets were split in half to a training and test part with 1000 subjects each. For all the scenarios, to account for the whole trajectory of the biomarker, the joint model, which consists of the submodels shown in Section 5.1, was fitted to the part of the simulated data sets that were kept for training. On the other hand, for the extrapolation method, the observations after reintervention were omitted from the analysis of the longitudinal outcome and the following mixed‐effects model was fitted to the data up to reintervention time:

$$y_{i}(t)=\eta_{i}(t)+\epsilon_{i}(t)=(\beta_{0}+b_{i0})+(\beta_{1}+b_{i1})t_{i}+\epsilon_{i}(t).$$

While for the survival process, the same model was used. For the time‐dependent Cox model, no longitudinal model was used and we only accounted for a change in the instantaneous hazard after the occurrence of the intermediate event as discussed in the previous section.

To assess the performance of the three approaches, we used the models that were fitted on the training data to calculate the time‐dependent AUCs and the PEs based on the test data. Both the time‐dependent AUCs and the PEs were calculated at three different time intervals starting at *t*=20, *t*=22, and *t*=24, respectively, and assuming a clinically relevant time interval of 2 years, Δ*t*=2. The time intervals were selected on the basis of when the most events occur in the simulated data.

**Figure 6:**
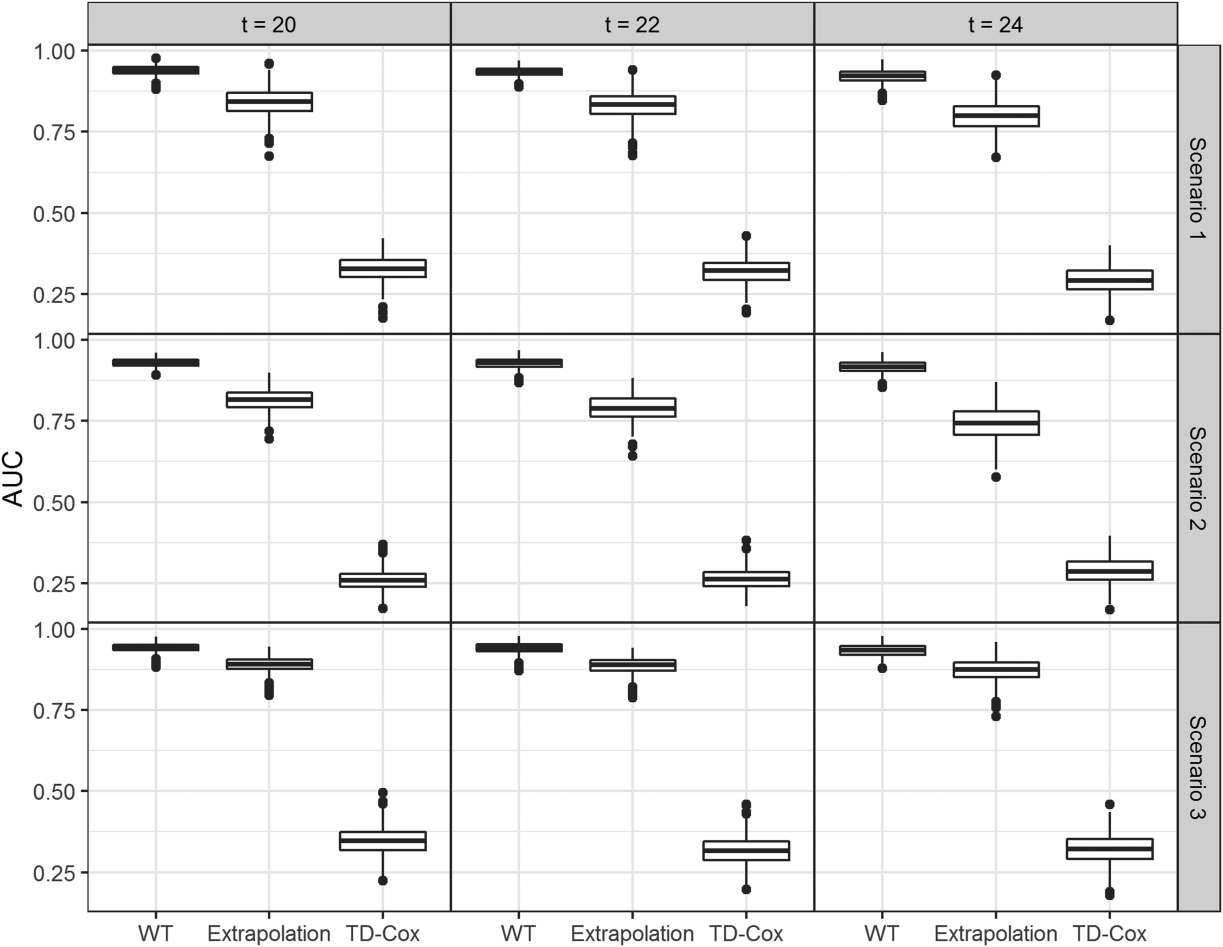
Area under the receiver operating characteristic curves (AUCs) for the individualized dynamic predictions, evaluated using the testing part of the 500 data sets for two different joint models. (1) Extrapolation: Assuming that the longitudinal profile does not change after the occurrence of the intermediate event; (2) Whole trajectory (WT): Assuming that the longitudinal profile changes after the occurrence of the intermediate event

**Figure 7:**
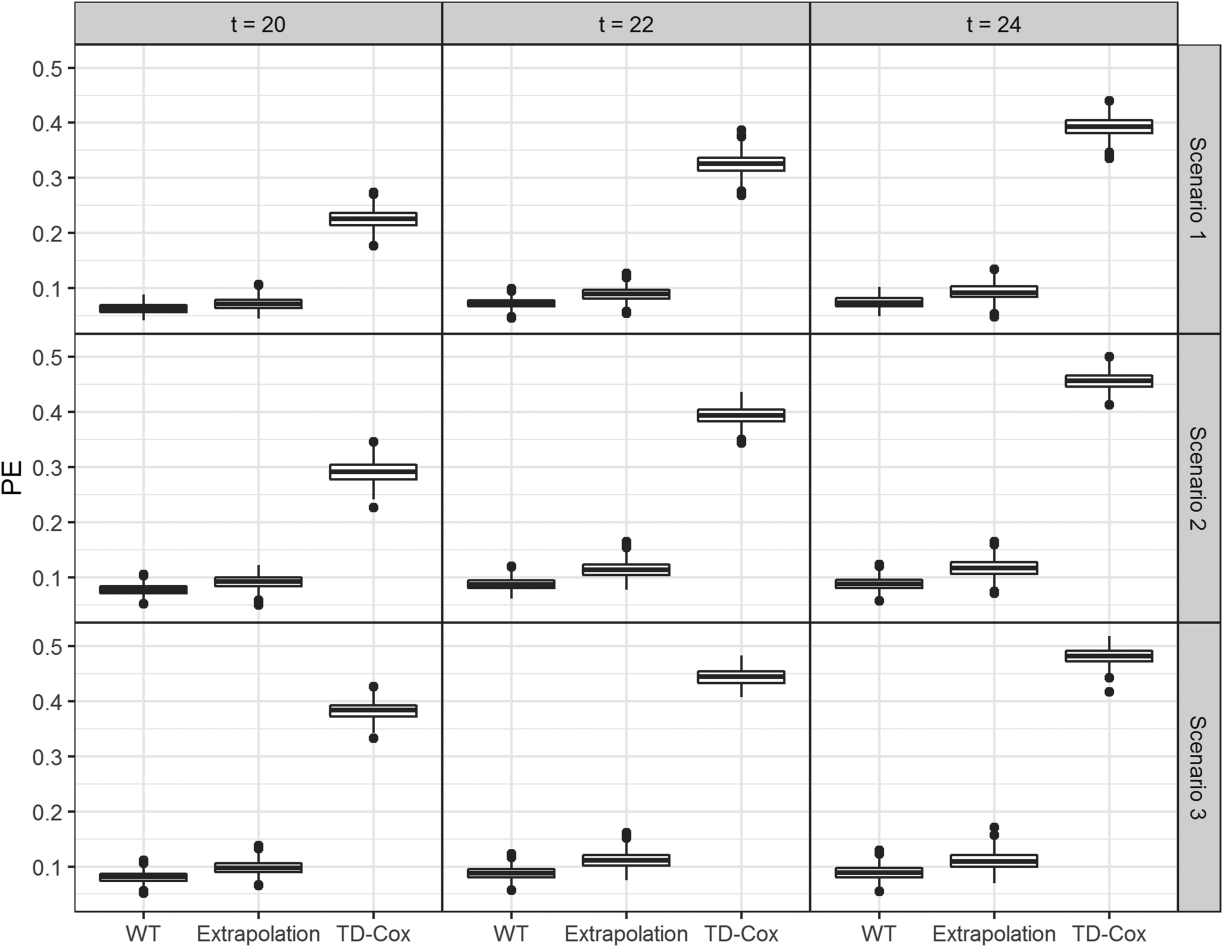
Prediction errors (PEs) for the individualized dynamic predictions, evaluated using the testing part of the 500 data sets for two different joint models. (1) Extrapolation: Assuming that the longitudinal profile does not change after the occurrence of the intermediate event; (2) Whole trajectory (WT): Assuming that the longitudinal profile changes after the occurrence of the intermediate event

In Figures 6 and 7, we present the results of the simulation study depicted by boxplots. Specifically, the boxplots in each row represent different scenarios, ie, nonzero effects, zero change in slope, and zero “drop” at time of reintervention and in each column a different time interval for prediction. In all the scenarios and time intervals, both the AUC and PE are better when assuming that the intermediate event changes both the risk for the event of interest and the longitudinal trajectory. The simple time‐dependent Cox model performs considerably worse than the other approaches. Moreover, there is a slight increase in the difference between the WT and extrapolation methods for both predictive measures as the time interval is set at later time points. That is, the more information used, the greater the difference between the two methods tends to become. Moreover, the relative performance of the two approaches does not differ between the three scenarios as well as for the different follow‐up times. Therefore, the results support the argument that accounting for the changes in the longitudinal trajectories due to the occurrence of the intermediate event improve the predictive accuracy when compared to the approach that the longitudinal profile remains unaffected by its occurrence.

## 6 DISCUSSION

Using the joint modeling framework, we developed tools for deriving individualized dynamic predictions that are adaptive to different scenarios regarding intermediate events, such as treatment changes or the occurrence of adverse events. We proposed a range of joint models for longitudinal and time‐to‐event data, which can accommodate special features due to the occurrence of intermediate events in both the longitudinal and survival submodels. That is, by incorporating features, such as the ones described in (1) and (2), a broad range of flexible joint models is sketched which accounts for the impact of an intermediate event by allowing for (1) a direct effect of the intermediate event on the risk of the clinical endpoint through the time‐varying binary covariate *R*_*i*_(*t*), (2) a direct effect of the intermediate event on the longitudinal trajectory through *g*{*R*_*i*_(*t*),*t*_*i*+_}, and (3) an indirect effect of the intermediate event on the risk of the clinical endpoint through the association between the two outcomes, which is defined by $f_{\left(t,\rho_{i}\right)}\{(H_{i}(t,\rho_{i}),b_{i}\}$ and is allowed to differ before and after the intermediate event. All these features allow for great flexibility in the specification of the joint model, which, when utilized accordingly, can lead to accurate predictions.

In the same line as recent observations with regard to dynamic predictions from joint models, we have seen that the accuracy of the predictions is influenced by intermediate events occurring during follow‐up. Such events will need to be appropriately modeled as time‐varying covariates in both the longitudinal and survival submodels. As illustrated in our simulation study, doing so, improves the predictive accuracy of the individualized dynamic predictions.

The focus of the applications presented in this paper was to illustrate how joint models can be utilized to provide individualized dynamic predictions under different scenarios with respect to the occurrence of intermediate events. It should be noted, however, that in the analysis of the pulmonary gradient data set, presented in Section 4.1, models M1, M2, and M3 assume that reoperation has a proportional effect on the hazard at any time point. This is a strong assumption since clinically it would make sense to assume that reoperation increases the hazard shortly after its occurrence before its beneficial effect takes place. However, model diagnostics based on Schoenfeld residuals did not point to a violation of this assumption. Since the focus of this application was to illustrate the exploitation of joint models in deriving individualized dynamic predictions, which are adaptive to different scenarios with respect to the occurrence of reoperation, we did not further explore the impact of this assumption. We do believe, however, that a more flexible model, such as a joint model that considers a multistate survival process with reoperation as a potential state would allow for such assumptions to be incorporated, which sets a potential direction for further research. Furthermore, it should also be noted that potential interaction effects between time and baseline characteristics such as age and sex were not explored in the modeling of the pulmonary gradient for parsimony, but they could be added.

It is also essential to discuss to what extent the proposed models can be used to draw inference on potential outcomes following different scenarios on the future occurrence of intermediate events. It is, therefore, crucial to note that the focus of this paper was prognostic modeling and the development of a prediction tool that can quantify potential changes in the outcome under different scenarios concerning the occurrence of future intermediate events. Consequently, the adaptive individualized prediction tools we developed can be used to answer questions such as “what is the expected change in the survival probability of a patient if he/she gets reoperated in a year from now given the information available on the patient so far?”. Such questions should not be confused with inferential statements of causal nature such as “A reoperation on this patient a year from now will cause his/her risk to change by this quantity.” For such statements to be possible, a set of essential assumptions including positivity, consistency and exchangeability should be satisfied, similar to the assumptions that any statistical model must fulfill to be used for causal inference. An informative reference on the topic, specifically for the case of longitudinal data and time‐varying treatments can be found in chapters 19 to 21 in the work of Hernán and Robins.^17^

Another interesting point of discussion for such applications is whether the proposed models are susceptible to time‐dependent confounding. Indeed, in the framework of a randomized clinical trial, the target treatment effect would no longer be protected if a specific selection of patients (eg, severe cases) experienced the intermediate event postrandomization. This complicates the interpretation of the treatment effect coefficient. Therefore, it should be noted that, in the applications presented, in this paper, we worked under the assumption that the intermediate event depends only on previous measurements of the marker and does not carry any further information. Under this assumption, including the intermediate event in the specification of both the mixed‐effect submodel and the relative risk submodel is sufficient and the process that generates the intermediate event does not have to be explicitly modeled.

The joint model formulation we presented allows to utilize the quantification of the effects the intermediate event imposes on the risk for the clinical endpoint of interest. As such, it can be utilized to derive individualized dynamic predictions for new subjects who did not experience the intermediate event and quantify how its occurrence at any future time point will influence their risk predictions. Such a predictive tool can provide valuable information to the physicians and assist in their decision‐making process for potential treatment changes. Based on such predictions, further prognostic tools can potentially be developed. For example, in settings where the timing of a future treatment is important, having both benefits and disadvantages when applied either too late or too early, such dynamic predictions that are adaptive to the timing of the intermediate event can become the basis for methodology that can be used to predict the optimal time for the future treatment. Unfortunately, the applications at hand did not allow for exploring such a possibility, but this is a clear direction for future research. Moreover, in our paper, we only consider the joint analysis of one longitudinal and one survival outcome. While the extension of the proposed models to their multivariate counterparts is straightforward, such multivariate joint models have not been explored in the literature in the context of intermediate events that may occur during follow‐up and alter the course of the disease for the patient. Therefore, individualized dynamic predictions based on even more complex joint models, such as with multiple longitudinal biomarkers or with multistate processes instead of a single time‐to‐event outcome might lead to improved accuracy depending on the application.

## Supporting information

SIM_8387‐Supp‐0001‐Supplementary_Material (SIM‐18‐0316_R2).pdf
