# Supplementary material for: Individualized dynamic prediction of survival with the presence of intermediate events
Source: Stat Med. 2019 Oct 30;38(30):5623–40. doi: 10.1002/sim.8387 (PMC6916395; doi:10.1002/sim.8387)
Supplement: Supplementary file 1 — SIM_8387‐Supp‐0001‐Supplementary_Material (SIM‐18‐0316_R2).pdf [file SIM-38-5623-s001.pdf]

Supplementary Material for “Individualized Dynamic Prediction of Survival with Intermediate Events”

Grigorios Papageorgiou MSc[1,2], Mostafa M. Mokhles MD PhD[2], Johanna J.M. Takkenebrg MD PhD[2], Dimitris Rizopoulos PhD[1]

[1]Department of Biostatistics, Erasmus University Medical Centre, Rotterdam, 3015 CN, the Netherlands
[2]Department of Cardiothoracic Surgery, Erasmus University Medical Centre, Rotterdam, 3000 CA, the Netherlands

S1 Data Description

S1.1 Pulmonary Gradient Data

Table S1.1.1: Data description: Continuous variables are presented as median (IQR), categorical variables as counts (frequencies %).

	All	Non reoperated	Reoperated
Number of subjects	467	402 (86.9%)	65 (13.1%)
Number of repeated measurements	8 (5 - 12)	7 (4 - 11)	14 (11 - 16)
Number of Events	34 (7.3%)	32 (8.0%)	2 (3.0 %)
Sex = Male	272 (58.2%)	229 (57.0%)	43 (66.1 %)
Age	20.3 (9.5 - 30.9)	21.5 (9.4 - 31.2)	16 (10.9 - 22.4)

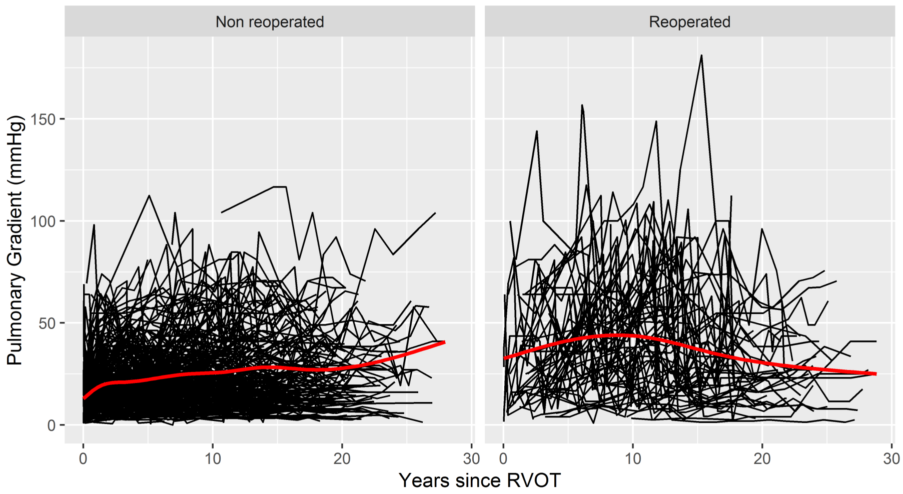

Figure S1.1.1: Individual trajectories of observed pulmonary gradient values over time for non reoperated subjects (left panel) and reoperated subjects (right panel) along with a smooth curve (red line).

S1.2 SPRINT Data

Table S1.2.1: Data description: Continuous variables are presented as median (IQR), categorical variables as counts (frequencies %).

	All	No SAE	SAE
Number of subjects	9068	5644 (62.2%)	3424 (37.8%)
Number of repeated measurements	15 (13 - 17)	15 (13 - 17)	14 (12 - 17)
Number of Events	535 (5.9%)	20 (0.3%)	515 (15.0 %)
Treatment = Intensive	4552 (50.2%)	2804 (49.7%)	1748 (51.0%)

S2 Predictive performance comparison between extrapolation method and joint models with intermediate events in the SPRINT data

To illustrate the use of the predictive performance measures presented in Section 3, we hereby present a comparison in terms of the time-dependent area under the receiver operating characteristic curve (AUC) and the expected prediction error (PE) for the SPRINT data.

More specifically, we are interested to compare the joint model used for the analysis of the SPRINT data which postulates effects of both the current value and current slope of the systolic blood pressure trajectory on the instantaneous risk of the composite endpoint (as presented in Section 4.2) against the corresponding model that ignores the longitudinal data after the occurrence of the serious adverse event (Extrapolation method). Specifically, for the extrapolation method the following model was used for the evolution of the systolic blood pressure:

$$SBP_i\left(t\right) = \left(\beta_0 + b_{i0}\right) + \left(\sum_{k=0}^{3} \left(\beta_{(k+1)} + b_{i(k+1)}\right) B_k\left(t,k\right)\right) + \beta_5 \text{Treatment}_i + \left(\sum_{k=0}^{3} \beta_{(k+6)} B_k\left(t,k\right)\right) \times \text{Treatment}_i + \epsilon_i\left(t\right),$$

while for the instantaneous risk the same model was used for both methods.

To compare the two approaches, we randomly split the data in half to a training and test part. Then we fitted the models to the training part and evaluated the AUC and PE for both models using the test part. The evaluation was done at 6 time-intervals: $t = 2.5$, $t = 2.75$, $t = 3$, $t = 3.25$, $t = 3.5$, and $t = 3.75$ assuming a clinically relevant time interval of a quarter of a year $\Delta t = 0.25$.

The results are shown in table **S3.7**. In the specific application the two approaches are virtually similar. This can be explained by the fact that the occurrence of a serious adverse event did not have a strong effect on the evolution of systolic blood pressure. However, even in this case both approaches achieve similar performance in terms of predictive accuracy.

Table S2.1: Evaluation of Predictive Performance between WT and Extrapolation Methods for the SPRINT data

	WT		Extrapolation	
t	AUC	PE	AUC	PE
2.50	0.66088	0.00362	0.65809	0.00363
2.75	0.79924	0.00417	0.79290	0.00418
3.00	0.54684	0.00606	0.56923	0.00607
3.25	0.42924	0.00368	0.46713	0.00369
3.50	0.62177	0.00421	0.63544	0.00423
3.75	0.68023	0.00570	0.66712	0.00571

S3 Sample R Code

In this section we provide a sample **R** code for fitting the joint models proposed in the paper and then utilizing them to derive predictions under different scenarios for the occurrence of intermediate events. As an illustrative example we use one of the 500 datasets generated for the simulation study,

presented in the paper, under scenario 1. Under this scenario we assume that the trajectory of the longitudinal outcome immediately changes (drops) at the occurrence of the intermediate event while the rate of increase (slope) also changes after the occurrence of the intermediate event, implying two special features of the longitudinal outcome that need to be considered.

To start with the code, the **R** package **JMbayes** needs to be installed and loaded.

```
# Install package JMbayes
install.packages("JMbayes")
# or for the latest development version of JMbayes
# Note that package devtools needs to be installed and loaded
install_github("drizopoulos\JMbayes")
```

For the installation of **JMbayes** we do recommend the second option (via github) since it is frequently updated.

Before we begin fitting the models it is important to have a look in the dataset, since it needs to be appropriately prepared before we use it. Table **S3.1** shows the data from two subjects in the dataset, one who experienced the intermediate event during follow-up and one that did not. The data are in long format and the following information are given:

- `ID`: Subject's identification number.
- `time`: Time the measurement of the longitudinal outcome was obtained.
- `Y`: Observed longitudinal outcome.
- `Interm.Evnt.Time`: Time the intermediate event occurred.
- `Tstart`: Starting time point for interval regarding the survival outcome in counting process format.
- `Tstop`: Stop time point for interval regarding the survival outcome in counting process format.
- `Event`: Event indicator corresponding to the interval defined by `Tstart` and `Tstop`.

Table S3.1: Example Dataset

ID	time	Y	Interm.Evnt.Time	Tstart	Tstop	Event
1	0.000	22.043	12.846	0.000	1.179	0
1	1.179	20.319	12.846	1.179	4.793	0
1	4.793	23.387	12.846	4.793	8.202	0
1	8.202	26.034	12.846	8.202	9.105	0
1	9.105	24.857	12.846	9.105	12.846	0
1	12.846	27.046	12.846	12.846	16.821	0
1	16.821	17.251	12.846	16.821	17.200	0
1	17.200	18.261	12.846	17.200	17.223	0
1	17.223	19.069	12.846	17.223	18.753	0
2	0.000	23.424	NA	0.000	2.401	0
2	2.401	24.342	NA	2.401	4.210	0
2	4.210	26.170	NA	4.210	4.451	0
2	4.451	25.781	NA	4.451	6.419	0
2	6.419	29.663	NA	6.419	7.265	1

In Table **S3.1** it is important to note that for subject 2, for which the intermediate event did not occur, data on the timing of the occurrence of the intermediate event are most likely to appear as missing observations in real life datasets. This causes a practical issue since in order to account for the special features, the occurrence of the intermediate event imposes on the longitudinal trajectory, we need to introduce appropriate time-varying covariates in the dataset, such as $R_{(t)}$ and t_{i+} which were discussed in Section 2 of the manuscript. That is we need to use the information we have regarding the timing of the occurrence of the intermediate event in creating these new variables, but doing so using missing observations will also result in missing observations for the new variables. To surpass this, we propose to change the missing observations for the timing of the occurrence of the intermediate event to arbitrarily large values. That is:

```
# For the remainder the R symbol 'dat' will be used to refer to the dataset
# Replace NAs for Interm.Evnt.Time with 1e+6
dat$Interm.Evnt.Time <- ifesle(is.na(dat$Interm.Evnt.Time), 1e+5, dat$Interm.Evnt.Time)
```

Here we chose a large unrealistic value of 100000 years that is impossible to occur in reality. Table **S3.2** shows how the data for the 2nd subject changed.

Table S3.2: Example Dataset

ID	time	Y	Interm.Evnt.Time	Tstart	Tstop	Event
1	0.000	22.043	12.846	0.000	1.179	0
1	1.179	20.319	12.846	1.179	4.793	0
1	4.793	23.387	12.846	4.793	8.202	0
1	8.202	26.034	12.846	8.202	9.105	0
1	9.105	24.857	12.846	9.105	12.846	0
1	12.846	27.046	12.846	12.846	16.821	0
1	16.821	17.251	12.846	16.821	17.200	0
1	17.200	18.261	12.846	17.200	17.223	0
1	17.223	19.069	12.846	17.223	18.753	0
2	0.000	23.424	100000.000	0.000	2.401	0
2	2.401	24.342	100000.000	2.401	4.210	0
2	4.210	26.170	100000.000	4.210	4.451	0
2	4.451	25.781	100000.000	4.451	6.419	0
2	6.419	29.663	100000.000	6.419	7.265	1

Now we can proceed to compute and include in the dataset the time-varying covariates of interest: $R_{(t)}$ and t_{i+} which correspond to the inermediate event indicator and the time relative to the occurrence of the intermediate event, respectively. To do so in **R** we run the following code:

```
# Compute the intermediate event indicator
dat$Int.Evnt.Index <- as.numeric(dat$time >= dat$Interm.Evnt.Time)
# Compute Time relative to the intermediate event
dat$time.relative <- pmax(0, dat$time - dat$Interm.Evnt.Time)
```

Table **S3.3** shows the updated data on the subset of the data that includes the two subjects we use in the example. We see that the two time-varying covariates are now included in the dataset. For subject 1, for all the time points following the occurrence of the intermediate event, the two time-varying covariates change accordingly whereas for subject 2 they are zero since the occurrence of the intermediate event was not observed during follow-up.

Table S3.3: Example Dataset

ID	time	Y	Interm.Evnt.Time	Tstart	Tstop	Event	Int.Evnt.Index	time.relative
1	0.000	22.043	12.846	0.000	1.179	0	0	0.000
1	1.179	20.319	12.846	1.179	4.793	0	0	0.000
1	4.793	23.387	12.846	4.793	8.202	0	0	0.000
1	8.202	26.034	12.846	8.202	9.105	0	0	0.000
1	9.105	24.857	12.846	9.105	12.846	0	0	0.000
1	12.846	27.046	12.846	12.846	16.821	0	1	0.000
1	16.821	17.251	12.846	16.821	17.200	0	1	3.975
1	17.200	18.261	12.846	17.200	17.223	0	1	4.354
1	17.223	19.069	12.846	17.223	18.753	0	1	4.377
2	0.000	23.424	100000.000	0.000	2.401	0	0	0.000
2	2.401	24.342	100000.000	2.401	4.210	0	0	0.000
2	4.210	26.170	100000.000	4.210	4.451	0	0	0.000
2	4.451	25.781	100000.000	4.451	6.419	0	0	0.000
2	6.419	29.663	100000.000	6.419	7.265	1	0	0.000

Now that the data are ready, we can proceed to fitting the joint model. This is no different than fitting any other joint model with package **JMbayes** apart from including in the model specification the newly introduced time-varying covariates. Hence we first fit separately the Cox relative risk and mixed-effects submodels and then we pass the corresponding objects to function `jointModelBayes()` to obtain the final joint model fit:

```
# Fit time-dependent Cox model
cox.fit <- coxph(Surv(Tstart, Tstop, Event) ~ Int.Evnt.Index + cluster(ID), data = dat)
# Fit the mixed-effects model
mix.fit <- lme(Y ~ time + Int.Evnt.Index + time.relative,
               random = ~ time + Int.Evnt.Index + time.relative | ID,
               data = dat)
# Fit the Joint model
joint.fit <- jointModelBayes(mix.fit, cox.fit, timeVar = "time")
```

After obtaining the joint model fit, we can proceed in deriving dynamic predictions under different scenarios for the occurrence of the intermediate event for new subjects. In Table **S3.4** the data for a new subject which has not experienced the intermediate event are shown.

Table S3.4: Example Dataset

ID	time	Y	Interm.Evnt.Time	Int.Evnt.Index	time.relative
New	0.5548873	15.86804	NA	NA	NA
New	1.3547850	26.81457	NA	NA	NA
New	2.1550550	13.02823	NA	NA	NA
New	6.5422016	29.46144	NA	NA	NA
New	7.5409739	23.48326	NA	NA	NA

For the code illustration this data are assumed to be included in a `data.frame` called `newdata`. Since this subject has not experienced the intermediate event, all the relevant information regarding the occurrence of the intermediate event are shown as missing observations. Again in order to derive predictions under different scenarios regarding the occurrence of the intermediate event we need to manipulate the data accordingly. We will illustrate how the data need to be changed for investigating three different scenarios regarding the occurrence of the intermediate event:

1. No occurrence of the intermediate event,
2. immediate occurrence of the intermediate event,
3. occurrence of the intermediate event after 1 year.

For the first case (no occurrence of the intermediate event) we will use the same trick as before. That is we will specify the time of the intermediate event as an arbitrarily large number and then calculate the intermediate event indicator and time relative to the occurrence of the intermediate event accordingly.

```
# Create a copy of newdata and call it newdata 1
newdata1 <- newdata
# Specify arbitrarily large number for Interm.Evnt.Time
newdata1$Interm.Evnt.Time <- 1e+05
# Compute Int.Evnt.Index and time.relative
newdata1$Int.Evnt.Index <-  as.numeric(newdata$time >= newdata$Interm.Evnt.Time)
newdata$time.relative <- pmax(0, newdata$time - newdata$Interm.Evnt.Time)
```

The changes in `newdata1` are shown in Table **S3.5**.

Table S3.5: Example Dataset

ID	time	Y	Interm.Evnt.Time	Int.Evnt.Index	time.relative
New	0.5548873	15.86804	1e+05	0	0
New	1.3547850	26.81457	1e+05	0	0
New	2.1550550	13.02823	1e+05	0	0
New	6.5422016	29.46144	1e+05	0	0
New	7.5409739	23.48326	1e+05	0	0

In a similar manner we create datasets `newdata2` and `newdata3` for scenarios 2 and 3 respectively. The only notable difference here, is that we add a new row in the dataset for which we only specify the covariates associated with time and the intermediate event accordingly but we specify the longitudinal outcome as missing for the specific row, since no data are available.

```
# DATA FOR SCENARIO 2
# create copy of newdata1 and call it newdata2
newdata2 <- newdata1
# Specify the time of the intermediate event to be equal to the last observed time point plus
# an arbitrarily small value
newdata2$Interm.Evnt.Time <- newdata2$time[nrow(newdata2)] + 0.00001
```

```
# create additional row
new.row <- newdata2[nrow(newdata2), ]
new.row$time <- newdata2$Interm.Evnt.Time[1]
new.row$Y <- NA
# attach new row to the dataset
newdata2 <- rbind(newdata2, new.row)
# compute time-varying covariates
newdata2$Int.Evnt.Index <- as.numeric(newdata2$time >= newdata2$Interm.Evnt.Time)
newdata2$time.relative <- pmax(0, newdata2$time - newdata2$Interm.Evnt.Time)

# DATA FOR SCENARIO 3
# create copy of newdata1 and call it newdata3
newdata3 <- newdata1
# Specify the time of the intermediate event to be equal to the last observed time point
# plus 1
newdata3$Interm.Evnt.Time <- newdata3$time[nrow(newdata3)] + 1
# create additional row
new.row <- newdata3[nrow(newdata3), ]
new.row$time <- newdata3$Interm.Evnt.Time[1]
new.row$Y <- NA
# attach new row to the dataset
newdata3 <- rbind(newdata3, new.row)
# compute time-varying covariates
newdata3$Int.Evnt.Index <- as.numeric(newdata3$time >= newdata3$Interm.Evnt.Time)
newdata3$time.relative <- pmax(0, newdata3$time - newdata3$Interm.Evnt.Time)
```

The changes in `newdata2` and `newdata3` are shown in Tables **S3.6** **S3.7**, respectively. Finally, the predictions under the different scenarios can be obtained by applying function `survfitJM()` to the fitted models and datasets we just created:

```
# Predictions for scenario 1
preds.1 <- survfitJM(object = joint.fit, newdata = newdata1, idVar = "ID")
# Predictions for scenario 2
preds.2 <- survfitJM(object = joint.fit, newdata = newdata2, idVar = "ID")
# Predictions for scenario 3
preds.3 <- survfitJM(object = joint.fit, newdata = newdata3, idVar = "ID")
```

Table S3.6: Example Dataset

ID	time	Y	Interm.Evnt.Time	Int.Evnt.Index	time.relative
New	0.5548873	15.86804	7.540984	0	0
New	1.3547850	26.81457	7.540984	0	0
New	2.1550550	13.02823	7.540984	0	0
New	6.5422016	29.46144	7.540984	0	0
New	7.5409739	23.48326	7.540984	0	0
New	7.5409839	NA	7.540984	1	0

Table S3.7: Example Dataset

ID	time	Y	Interm.Evnt.Time	Int.Evnt.Index	time.relative
New	0.5548873	15.86804	8.540974	0	0
New	1.3547850	26.81457	8.540974	0	0
New	2.1550550	13.02823	8.540974	0	0
New	6.5422016	29.46144	8.540974	0	0
New	7.5409739	23.48326	8.540974	0	0
New	8.5409739	NA	8.540974	1	0

S4 Data Availability Statement

The data used for the Pulmonary Gradient analysis are available on request from the corresponding author. The data are not publicly available due to privacy or ethical restrictions. The data used for the SPRINT trial are available from BioLINCC.
